# Hybridization Hotspots at Bat Swarming Sites

**DOI:** 10.1371/journal.pone.0053334

**Published:** 2012-12-28

**Authors:** Wiesław Bogdanowicz, Krzysztof Piksa, Anna Tereba

**Affiliations:** 1 Museum and Institute of Zoology, Polish Academy of Sciences, Warszawa, Poland; 2 Cracow Pedagogical University, Institute of Biology, Kraków, Poland; Texas A&M University, United States of America

## Abstract

During late summer and early autumn in temperate zones of the Northern Hemisphere, thousands of bats gather at caves, mainly for the purpose of mating. We demonstrated that this swarming behavior most probably leads not only to breeding among bats of the same species but also interbreeding between different species. Using 14 nuclear microsatellites and three different methods (the Bayesian assignment approaches of STRUCTURE and NEWHYBRIDS and a principal coordinate analysis of pairwise genetic distances), we analyzed 375 individuals belonging to three species of whiskered bats (genus *Myotis*) at swarming sites across their sympatric range in southern Poland. The overall hybridization rate varied from 3.2 to 7.2%. At the species level, depending on the method used, these values ranged from 2.1–4.6% in *M. mystacinus* and 3.0–3.7% in *M. brandtii* to 6.5–30.4% in *M. alcathoe*. Hybrids occurred in about half of the caves we studied. In all three species, the sex ratio of hybrids was biased towards males but the observed differences did not differ statistically from those noted at the population level. In our opinion, factors leading to the formation of these admixed individuals and their relatively high frequency are: i) swarming behaviour at swarming sites, where high numbers of bats belonging to several species meet; ii) male-biased sex ratio during the swarming period; iii) the fact that all these bats are generally polygynous. The highly different population sizes of different species at swarming sites may also play some role. Swarming sites may represent unique hybrid hotspots, which, as there are at least 2,000 caves in the Polish Carpathians alone, may occur on a massive scale not previously observed for any group of mammal species in the wild. Evidently, these sites should be treated as focal points for the conservation of biodiversity and evolutionary processes.

## Introduction

At least 25% of plant species and 10% of animal species are involved in hybridization and potential introgression with other species [1], [2]. This level is much lower in bats, with about 1,260 species known world-wide [3] and about 14 species known to produce interspecific hybrids. The few published cases include black (*Pteropus alecto*) and grey-headed (*P. poliocephalus*) flying-foxes [4], sibling species of the horseshoe bat (*Rhinolophus yunanensis* and *R. pearsoni* [5]), common (*Pipistrellus pipistrellus*) and soprano (*P. pygmaeus*) pipistrelles [6]), the mouse-eared bat (*Myotis myotis* and *M. oxygnathus* [7], [8], see also [9]), and the bent-winged bat (*Miniopterus schreibersii* and *M. pallidus* [10]). Hybrid origin of a Caribbean species of bat (*Artibeus schwartzi*) has also been hypothesized, where hybridization among three species and subsequent isolation of hybrids have contributed to the formation of a distinct species-level lineage [11]. Additionally, there is a growing but still low number of cases showing historical introgression events and a replacement of the mitochondrial genome in one species by another (e.g., [7], [12], [13], see also [14]).

Bat swarming is a complex phenomenon, and its causes have yet to be fully explained. The primary hypotheses, not being mutually exclusive, invoke mating behavior, information transfer regarding suitable hibernacula, and the use of caves as resting sites during seasonal migration (e.g., [15]–[17]). It occurs in late summer and autumn, and involves sustained chasing followed by copulation at underground sites. Mating activity in temperate bats can also take place in winter and spring, as suggested by the presence of sperm in the caudae epididymides in males of some species (e.g., [18], [19]), although it is usually of much lower intensity and scale than during the swarming period ([20], authors' own observations). From an evolutionary perspective swarming appears to promote gene flow among bat colonies, increasing genetic diversity and preventing inbreeding [21]–[24].

In our study, we assessed whether mating in swarming bats at swarming sites, where thousands of bats meet and males outnumber females [17], [23], [25], is directed exclusively towards conspecific individuals or may also include interspecific matings. Mating in temperate bats can (and does) happen during the winter but there is certainly a much higher likelihood of such hybridization events in swarming sites where there is a mixture of closely related species meeting primarily for the purpose of mating. We focused on three small (4–9 g) and morphologically very similar species of so-called whiskered bats in the family Vespertilionidae: *Myotis mystacinus*, *M. brandtii*, and *M. alcathoe*. The first two species are of predominantly boreal Palaearctic distribution [26], [27]. *Myotis alcathoe* was described in 2001 [28] and is relatively poorly known, but current information suggests that it has a wide European range (e.g., [29]–[33]).

## Materials and Methods

### Study Area and Materials

**Figure 1:**
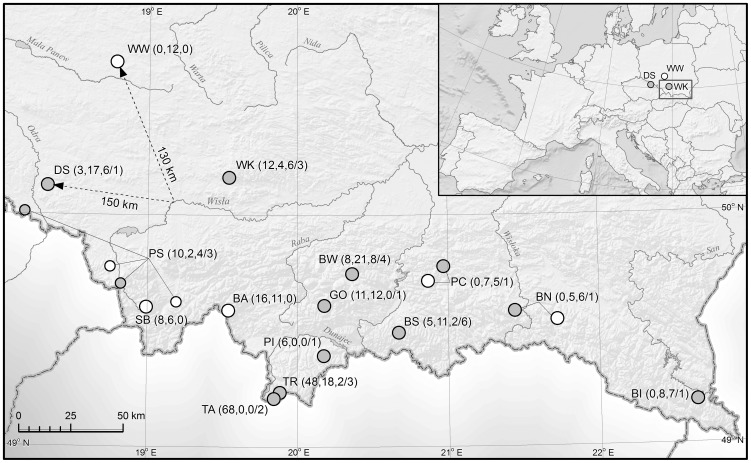
Location of Poland in Europe, study area (27 caves) in the southern part of the country, and the sites (in grey) where hybrids have been detected. The numbers of genotyped bats (*M. mystacinus*, *M. brandtii* and *M. alcathoe*, respectively) are given in parentheses, whereas the numbers of hybrids (as derived from STRUCTURE, with the 0.90 threshold) are presented after the dash. Sites are as follows: SB — Beskid Śląski Mountains (1 cave); BA — Babia Góra Mountain (1); TA — Tatra Mountains, caves at alpine zone (2); TR — Tatra Mountains, caves at forest zone (4); PI — Pieniny Mountains (1); GO — Gorce Mountains (1); BW — Beskid Wyspowy Mountains (2); BS — Beskid Sądecki Mountains (2); PC — Ciężkowickie Foothills (2); BN — Beskid Niski Mountains (2); BI — Bieszczady Mountains (1); PS — Silesian Foothill, Silesian Lowland and Żywiecka Valley (4); WK — Krakowska Upland (2); WW — Wieluńska Upland (1); DS — Sudety Mountains (1).

The study was performed at 27 caves in the southern mountainous part of Poland (see Figure 1). Bats were mist-netted outside cave entrances between July and October 2007–2010 [34]. Tissues for DNA extraction were taken using a 3-mm diameter wing membrane biopsy from the plagiopatagium. In total, we analyzed 195 samples of *M. mystacinus* (119 males and 76 females), 134 of *M. brandtii* (107 males and 27 females), and 46 of *M. alcathoe* (29 males and 17 females). Samples were stored in 85% ethanol. Morphological identification of all taxa (as defined in Table 1) was confirmed by sequencing a fragment of at least 500 bp of the mitochondrial ND1 gene (GenBank Accession Nos. JX645259–JX645319) [35].

**Table 1:** The list of morphological criteria used to discriminate among the three species of bats belonging to the *M. mystacinus* group [61], [62].

Feature	*M. alcathoe*	*M. mystacinus*	*M. brandtii*
Body size	1. The smallest species of the group.	1. Slightly larger than *M*. *alcathoe*, and similar or slightly smaller than *M*. *brandtii*.	1. Similar or slightly larger than *M*. *mystacinus*.
	2. The smallest forearm length, usually <33 mm (mean 32.32, s.d. 0.66, range 31.1–33.5).	2. The forearm length larger than in *M. alcathoe*, usually >33 mm (mean 34.81, s.d. 0.83, range 32.8–36.9).	2. The forearm length larger than in *M. alcathoe*, usually >34 mm (mean 35.65, s.d. 0.94, range 33.4–38.0).
	3. The smallest tibia length, usually <14.5 mm (mean 14.74, s.d. 0.88, range 13.2–16.1).	3. Tibia length as in *M. brandtii*, usually >15 mm (mean 15.37, s.d. 0.59, range 14.3–16.2).	3. Tibia length usually >15 mm (few measurements noted, only for the smallest individuals, all above 16 mm).
Body coloration	1. Coloration pattern of *M. alcathoe* close to *M. brandtii* (and *M. daubentonii*). Dorsal pelage brown, garish-brown or reddish brown; face and ears pale-colored.	1. Dorsal pelage very dark, frequently with yellowish tips giving bicolored appearance; face and ears dark brown to black.	1. Dorsal pelage with light-golden hair tips; skinny parts on the face and the base and inner part of ears pale (pinkish).
	2. Ears with lighter color inside.	2. Ears usually without lighter color inside.	2. Ears with lighter colour inside.
Tragus	1. Tragus short, not reaching the notch on the posterior edge of the ear or only scarcely.	1. Tragus as in *M. brandtii*, extending beyond the notch on the posterior edge of the ear.	1. Tragus extending beyond the notch on the posterior edge of the ear.
Penis morphology	1. Penis thin along its entire length (like in *M. mystacinus*).	1. Penis evenly narrow along its entire length (like in *M. alcathoe*).	1. Penis club-shaped at its end.
Dental morphology	1. Cingular cusp on P^4^ larger than in *M. mystacinus* although not so prominent as in *M*. *brandtii*.	1. The smallest cingular cusp on P^4^. 2nd premolars, P^3^ and P_3_ markedly smaller than the 1st ones, P^2^ and P_2_, respectively.	1. High cingular cusp on the last upper premolar (P^4^) which is equal in height or even higher than the second upper premolar (P^3^).

The forearm and tibia lengths shown in parentheses refer to those recorded in purebreds in the present study.

### Ethics Statement

All procedures were carried out under licenses from Ministry of Environment and from National Parks in Poland. Ministry of Environment specifically approved our study and provided licenses for collection of tissue samples. We also had permission to use the samples.

### Microsatellite Analysis

DNA was isolated from the tissue samples using the Genomic Mini Kit (A&A Biotechnology) according to the manufacturer's protocol. We amplified 14 nuclear microsatellite loci in four multiplexes [32], [36] using the Multiplex PCR Kit (Qiagen). PCR reactions were carried out in 15 µl reaction volumes with 10–50 ng DNA, 10 pmol of each primer, 7.5 µl Multiplex PCR Master Mix and 5 µl of PCR water. Each forward primer was labelled with fluorescent WellRED dyes (Beckman Coulter, Inc.). The PCR thermal profile followed the protocol used by Bogdanowicz *et al*. [35]. Allele lengths were scored on a CEQ 8000 sequencer (Beckmann-Coulter, Inc.) (see Appendix S1). We used MICRO-CHECKER [37] to test for scoring errors and null alleles.

### Genetic Differentiation and Identification of Hybrids

A principal coordinate analysis (PCA) of a pairwise, individual-by-individual genetic distance matrix calculated using the method of Huff *et al*. [38] implemented in GENEALEX ver. 6.1 [39] was used to visualize genetic structure in our sample, without any a priori grouping [39], [40]. Although PCA is not an ideal method to identify hybrids (i.e. there is no quantification of admixture), it can provide valuable information regarding divergence among major groups. In the case of well-separated groups, clustering of some individuals belonging to one species with the other species may indicate hybrid origin.

Two Bayesian clustering methods were used to identify hybrids: the approach implemented in STRUCTURE software ver. 2.3.3 [41] and that of NEWHYBRIDS, ver. 1.1 beta [42]. With STRUCTURE, we assumed that all three species contribute to the gene pool of the sample (*K* = 3), and looked at the proportion of an individual genotype originating from each of *K* categories. Runs were performed with a burn-in of 50,000 repetitions followed by 100,000 repetitions of sampling, and two iterations of each value of *K*. All runs were conducted assuming the admixture model and with allele frequencies correlated. Individuals were considered hybrids when their *q*-value of belonging to the putative species was lower than the given threshold (*Tq*) (see [43], [44]). Two threshold values were used to compare results: 0.75, which would correspond to an ideal backcross, and 0.90, a more restrictive one [43], [44]. In the NEWHYBRIDS model, the sample is taken from a mixture of pure individuals and hybrids. In this case, we used two of the three criteria described in Burgarella *et al.* [44] to estimate the hybrid proportion: the relatively relaxed 3rd criterion, when the threshold is applied only to the purebred category, and all individuals with *q*≥0.90 or 0.75 are considered purebred parentals and all others are considered hybrids (this is the only criterion where no individual remains unassigned); and the more strict 2nd criterion, in which all hybrid categories (F1, F2, and backcrosses) can be combined to identify admixed individuals without distinguishing hybrid categories [43], [44]. We omitted the most restrictive criterion (no. 1) where the threshold value is applied to each category separately because only 14 markers were used in the study, which is likely too few to confidently assign all categories. In NEWHYBRIDS we used Jeffrey's prior and a burn-in period of 50,000 repetitions followed by 100,000 repetitions of sampling. To rule out possible bias due to low frequencies of alleles an additional analysis with Uniform prior was carried out to check whether results are consistent [42]. To evaluate the diagnostic power of the markers used we calculated the allele frequency differential between species pairs (δ) [45]. For each locus, δ was calculated as a mean of half of the sum of absolute allele frequency differences. Values of δ≥0.5 indicate their high discriminatory power.

Additionally, we examined the possibility that the results obtained from the STRUCTURE and NEWHYBRIDS analyses could be observed by chance, and performed simulation studies following the protocol used by Burgarella *et al.* [44]. Based on allele frequencies calculated for parental species (without potential hybrids identified with *q*<0.90) we simulated 10,000 genotypes for each category (purebreds, F1, and backcrosses) with HYBRIDLAB 1.0 [46]. Genotypes were sampled without replacement with POPTOOLS 2.6 [47] to create a single sample set consisting of 400 individuals (and three species hybridizing simultaneously) with different hybrid proportions (HP = 0, 2.5, 7.0, and 10.0). For each HP, 20 replicates were generated. Sample sizes and HPs have been chosen to represent the actual population samples. In the above situation, however, all three species were selected randomly, without looking at their sample size ratio in the studied sample, and assuming symmetric hybridization rate. Such an approach may not match the observed data set and may be misleading (as one species may have much higher hybridization rate than others). To avoid this problem, we also performed simulations using sample sizes in the empirical data set and employing an asymmetric hybridization rate (see Table 2).

**Table 2:** Results of STRUCTURE and NEWHYBRIDS (2nd and 3rd criterion) analyses with simulated samples of *n* = 400.

							Power	Accuracy		
Simulated HP (%)	No. of repetitions	No. of hybrids in the sample	Method	*Tq*	Mean No. of hybrids (s.d.)	Estimated HP (%)	Hybrids	Purebreds	Hybrids	Purebreds	Type I error	Not assigned
0	20	0	STRUCTURE	0.90	0.30 (0.58)	0.087	-	0.999	-	1.000	0.001	
				0.75	0	0.000	-	1.000	-	1.000	0.000	
			NEWHYBRIDS	0.90	0	0.000	-	1.000	-	1.000	0.000	
				0.75	0.10 (0.30)	0.025	-	1.000	-	1.000	0.000	
0*	20	0	STRUCTURE	0.90	0.85 (0.87)	0.212	-	0.998	-	1.000	0.002	
				0.75	0	0.000	-	1.000	-	1.000	0.000	
			NEWHYBRIDS	0.90	0.05 (0.22)	0.012	-	1.000	-	1.000	0.000	
				0.75	0.05 (0.22)	0.012	-	1.000	-	1.000	0.000	
2.5	20	10	STRUCTURE	0.90	9.7 (0.80)	2.425	0.930	0.999	0.959	0.998	0.001	
				0.75	7.7 (1.12)	1.925	0.770	1.000	1.000	0.994	0.000	
			NEWHYBRIDS,	0.90	6.2 (1.76)	1.550	0.585	0.999	0.944	0.989	0.001	4
			2nd	0.75	6.5 (2.01)	1.625	0.590	0.998	0.908	0.990	0.002	2
			NEWHYBRIDS,	0.90	10.3 (4.30)	2.587	0.630	0.990	0.609	0.991	0.010	
			3rd	0.75	8.2 (3.27)	2.050	0.620	0.995	0.756	0.990	0.005	
				0.75	26.5 (3.61)	6.637	0.725	0.987	0.819	0.978	0.013	
7	20	28	STRUCTURE	0.90	27.1 (1.38)	6.787	0.970	1.000	1.000	0.998	0.000	
				0.75	23.1 (1.55)	5.775	0.807	0.999	0.978	0.986	0.001	
			NEWHYBRIDS,	0.90	18.8 (2.60)	4.712	0.661	0.999	0.981	0.975	0.001	8
			2nd	0.75	19.7 (2.97)	4.937	0.675	0.998	0.957	0.976	0.002	3
			NEWHYBRIDS,	0.90	27.1 (5.38)	6.775	0.700	0.980	0.723	0.977	0.020	
			3rd	0.75	22.8 (3.80)	5.700	0.691	0.991	0.849	0.977	0.009	
7*	20	28	STRUCTURE	0.90	27.4 (1.04)	6.850	0.954	0.998	0.974	0.977	0.002	
				0.75	23.2 (1.65)	5.812	0.830	1.000	1.000	0.987	0.000	
			NEWHYBRIDS,	0.90	15.4 (1.26)	3.837	0.541	0.999	0.987	0.967	0.001	2
			2nd	0.75	16.4 (1.34)	4.087	0.568	0.999	0.972	0.968	0.001	1
			NEWHYBRIDS,	0.90	18.9 (1.88)	4.725	0.591	0.994	0.876	0.970	0.006	
			3rd	0.75	17.4 (1.43)	4.350	0.577	0.997	0.928	0.969	0.003	
10	20	40	STRUCTURE	0.90	38.6 (1.53)	9.650	0.956	0.999	0.991	0.995	0.001	
				0.75	31.7 (2.17)	7.925	0.793	1.000	1.000	0.977	0.000	
			NEWHYBRIDS,	0.90	26.1 (5.02)	6.525	0.641	0.999	0.983	0.962	0.001	9
			2nd	0.75	27.2 (5.07)	6.787	0.656	0.998	0.967	0.963	0.003	3
			NEWHYBRIDS,	0.90	35.4 (6.69)	8.837	0.698	0.979	0.789	0.967	0.021	
			3rd	0.75	30.9 (5.84)	7.725	0.681	0.990	0.882	0.965	0.010	

Abbreviations: HP – hybrid proportion, *Tq* – threshold *q*-value. Power ( = efficiency sensu Vähä & Primmer [42]): number of correctly identified individuals for a category over the actual number of that category in the sample; accuracy: number of correctly identified individuals for a category over the total number of individuals assigned to that category [43], [44]. * denotes simulations conducted with samples sizes of the three species matching the empirical data set, and asymmetric hybridization rates consistent with those detected in the analysis of sampled data (as depicted in Fig. 3A).

## Results

**Figure 2:**
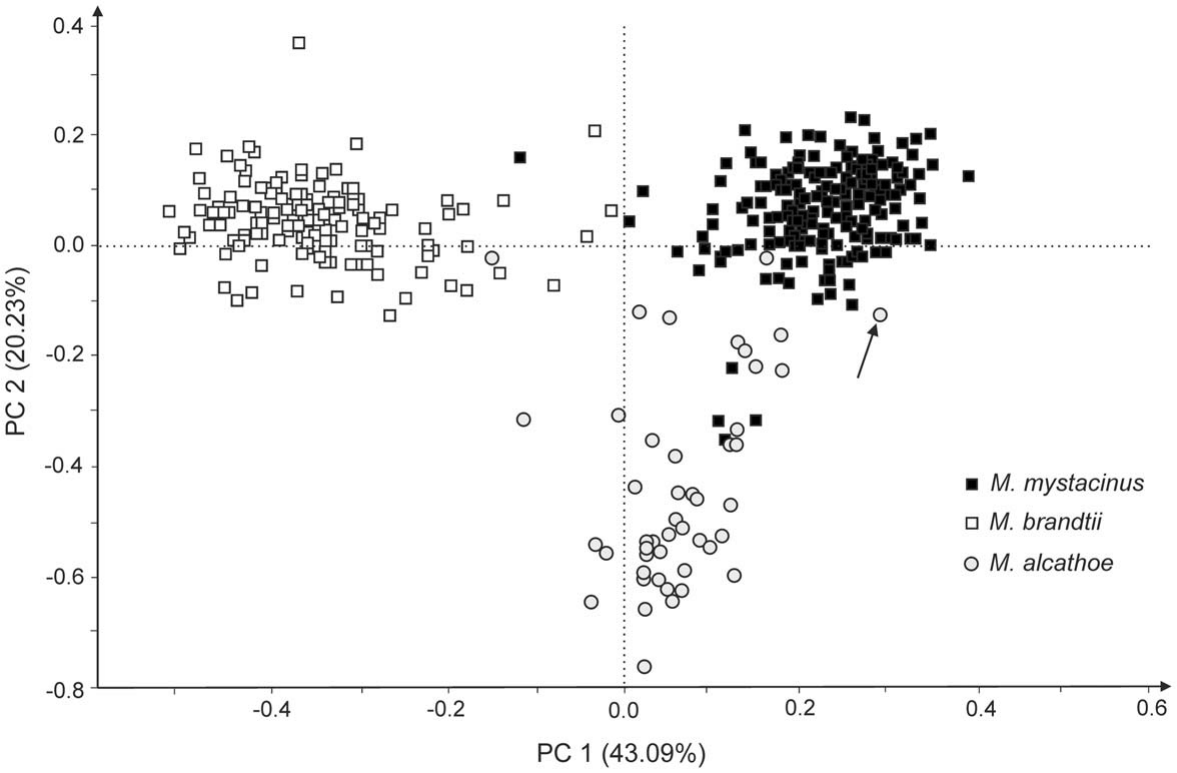
A two-dimensional plot of the principal coordinate analysis (PCA) performed using GENEALEX based on 14 microsatellite loci for *M. mystacinus*, *M. brandtii* and *M. alcathoe* (the percentage of variance for a given PC shown in parentheses). ID codes for particular species are based on mtDNA determination (in 5 males and 1 female of *M. brandtii*, 2 males of *M. mystacinus*, and 1 female of *M. alcathoe* – with an unsuccessful genetic sequencing – only morphological criteria were used; all of them are placed inside their parental groups). The arrow indicates individual no. BW_M01511 (see the results).

To check the possible interspecific gene exchange among three species, we analyzed 375 individuals originating from across their sympatric range in southern Poland (Figure 1) using biparentally inherited markers (14 microsatellite loci). In terms of genetic structure, there was a relatively clear division at the species level (Figure 2). Nevertheless, the overlap observed in the available data from the nuclear genome, with respect to mitochondrial identifications of each species, also suggests some level of mitochondrial introgression and reinforces the hypothesis of hybridization. Based on Figure 2, it seems that *M. alcathoe* mtDNA is introgressed into the genome of *M. mystacinus*, and there is one case of introgression (individual No. BW_M01511) into the *M. brandtii* gene pool. *Myotis mystacinus* shows introgression into both species. There is no evidence of mtDNA introgression from *M. brandtii* into either of the other two species, suggesting *M. brandtii* females may not be able to produce viable hybrids with *M. mystacinus* or *M. alcathoe*.

In the simulations, when the three species were sampled randomly (as opposed to sampling according to the ratios in the empirical data set) and we assumed a symmetric hybridization rate, both STRUCTURE and NEWHYBRIDS demonstrated a similar percentage of admixed individuals for both threshold values (Table 2). Nevertheless, when no hybrids were assumed, NEWHYBRIDS performed slightly better than STRUCTURE, which resulted in a small proportion of false hybrids with the 0.90 threshold option. On the contrary, when the simulated sample contained hybrids, the best hybrid proportion estimates were achieved with STRUCTURE and NEWHYBRIDS, 3rd criterion, and the threshold of 0.90. The power to correctly classify purebreds was higher than 97% in all approaches used, but STRUCTURE showed the highest power to detect true hybrids for both *Tq* levels. NEWHYBRIDS also left some individuals unassigned because of too low ability to detect them (criterion 2) or provided the highest number of wrongly assigned hybrids (criterion 3).

**Figure 3:**
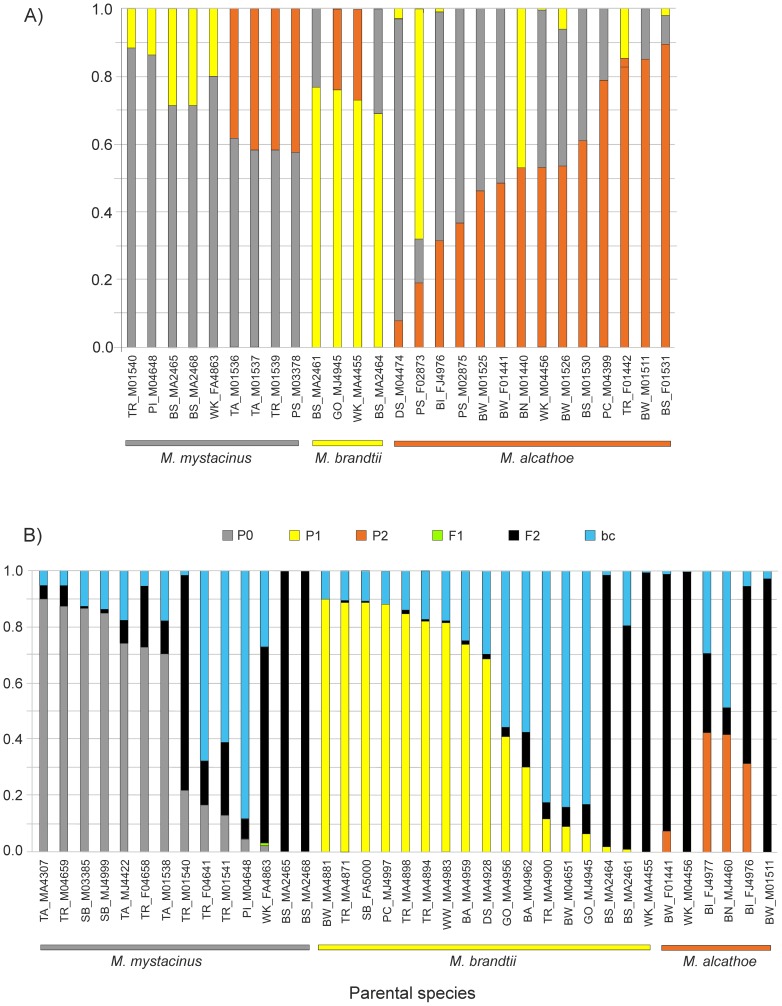
Posterior probability (*q*) for all individuals identified as putative hybrids by at least one of the method-threshold (*Tq*) combinations. Each individual is represented by a vertical bar partitioned into coloured segments. The length of each segment corresponds to (A) the membership proportions of each parental species estimated by STRUCTURE (*Tq*≥0.90) and (B) the probability of belonging to the parental species and the three hybrid classes (F1, F2, and first backcross with each of the parental (P0, P1, and P2) species) estimated by NEWHYBRIDS, criterion 3 (with the 0.90 threshold). Species are assigned according to their mtDNA ID. Individuals are identified by the swarming site code (see Figure 1), followed by sex (M — male, F — female), age (A — adult, J — juvenile, 0 — indetermined), and ID number.

Results were different when simulations were done using sample sizes proportional to the frequency of these species in the empirical data set and employing an asymmetric hybridization rate consistent with the results presented in Figure 3A. When no hybrids were assumed, both programs performed similarly well (although, as previously, NEWHYBRIDS was slightly better than STRUCTURE). However, when the different ratio of hybrid individuals was taken into account NEWHYBRIDS failed to detect F1 hybrids between *M. alcathoe* and *M. mystacinus* in all of the repetitions. This lead to low (<0.60) power to detect hybrids with the use of this software (Table 2). In contrast to NEWHYBRIDS, STRUCTURE showed moderate-to-high power (>0.80 for *Tq* = 0.75 and >0.95 for *Tq* = 0.90) to identify admixed individuals depending on the threshold value used.

Another question is if our microsatellite markers provide sufficient resolution to discriminate species and identify hybrids. Using a Bayesian assignment approach, errors can be expected and are dependent upon the number and frequency of shared alleles (e.g., STRUCTURE detected hybrids (although very few) when *Tq* = 0.9 and the proportion of simulated hybrids (HP = 0 and 0*) was zero – see Table 2). The more similarity in frequency of alleles among species, the higher error rate. However, all but two of the 14 markers used in the present study showed high allele frequency differential, δ≥0.5, indicating they possessed high discriminatory power (Table 3).

**Table 3:** Allele frequency differential (δ) between species pairs and the mean value for each locus arranged in decreasing order of mean δ.

	Species pair	
Locus	*mystacinus* vs. *brandtii*	*brandtii* vs. *alcathoe*	*mystacinus* vs. *alcathoe*	Mean
D15-Mluc	0.94	0.93	0.77	0.880
G2-Mluc	0.88	0.89	0.72	0.830
D15	0.99	0.89	0.48	0.786
B8-Mluc	0.78	0.95	0.54	0.756
G6-Mluc	0.84	0.78	0.65	0.754
EF15-Mluc	0.35	0.78	0.67	0.603
H23-Mluc	0.35	0.72	0.73	0.601
H29	0.41	0.71	0.67	0.597
G30	0.63	0.59	0.49	0.570
F19-Mluc	0.64	0.40	0.66	0.565
D9	0.40	0.71	0.48	0.532
G30-Mluc	0.57	0.53	0.45	0.517
F19	0.56	0.40	0.54	0.499
G31-Mluc	0.33	0.36	0.49	0.392

Results from the STRUCTURE analysis showed a surprisingly low probability of assignment (*q_i_*) in 27 individuals (Figure 3A): 9 identified initially as *M. mystacinus* (*q_i_* = 0.57–0.88), 4 as *M. brandtii* (*q_i_* = 0.69*–*0.77) and 14 as *M. alcathoe* (*q_i_* = 0.07–0.89). One case of ‘pure’ introgression involving *M. alcathoe* (BW_F01524; *q_i_* = 0.04) into the *M. mystacinus* (*q_i_* = 0.96) gene pool was also detected. Results from NEWHYBRIDS confirmed the existence of hybrid individuals. According to the most relaxed criterion (No. 3), there were 37 hybrids with the threshold *q-*value (*Tq*) at the level of 0.90 or 26 hybrids with *Tq* = 0.75 (Figure 3B). Even when the more strict assumptions were taken into account (criterion 2), there were still either 12 (9 F2 and 3 backcrosses; 4 *M. mystacinus*, 5 *M. brandtii*, and 3 *M. alcathoe*) or 16 (10 F2 and 6 backcrosses; 7 *M. mystacinus*, 6 *M. brandtii*, and 3 *M. alcathoe*) hybrids present in our data set. At the species level, backcrosses clearly prevailed in *M. brandtii*, whereas F2 hybrids formed a significant portion of hybrid individuals in *M. mystacinus* and *M. alcathoe* (Figure 3B).

Morphologically, the majority (ca. 90%) of hybrids detected based on nuclear DNA follow their mtDNA identification. This is especially clear in the case of males of *M. brandtii*, which are easily identified to species because of their characteristic penis, which is distinctly thickened at the end. A few individuals possessing classical morphology (i.e., shape and size) showed pelage coloration characteristic for other species, e.g., three *M. alcathoe* (DS_M04474, TR_F01442, and BS_F01531) had fur, ears, and wings of dark color characteristic of *M. mystacinus*. The opposite cases have also been reported, e.g., an individual identified as *M. mystacinus* (PI_M04648) possessed pelage color characteristic of its parental species and forearm length (30.9 mm) characteristic of *M. alcathoe*. Evidently, in the case of hybrids of these cryptic species, there is no unambiguous match between mtDNA IDs and phenotypes, and identification based on morphology (with the exception of male *M. brandtii*) may be misleading.

## Discussion

In our study, regardless of the model specifications, admixed individuals were detected in all three species examined. In terms of the number of hybrids, there was relatively good congruence between results from STRUCTURE (the highest power and accuracy) and the 2nd criterion of NEWHYBRIDS (the most restrictive) with thresholds of 0.90. Both methods showed a low probability of assignment for parental species in at least 3.2–7.2% of examined individuals. At the species level, these values were within the range of 2.1–4.6%, 3.0–3.7%, and 6.5–30.4% in *M. mystacinus*, *M. brandtii*, and *M. alcathoe*, respectively (Figure 3). These ranges, regardless of the method applied, are similar in the first two species but evidently differ in the case of *M. alcathoe* being almost 5 times higher when STRUCTURE is used. Nevertheless, this figure (30.4%), although unexpectedly high (e.g., in two cryptic species of pipistrelle bat the maximum hybridization rate varied from 11.1 to 13.3% [6]), appears to be closer to reality (at least locally) than 6.5% suggested by the NEWHYBRIDS approach. As seen in our simulation studies, in the case of our empirical data set, STRUCTURE outperforms NEWHYBRIDS, which underestimates the number of admixed individuals (see Table 2).

Jan *et al.* [32] also noted one individual of *M. mystacinus* with a low assignment (*q_i_* = 0.88), and suggested the possibility of either hybridization or an origin of this individual from an unsampled population. On the other hand, we did not discover any first generation (F1) hybrids. As documented by Vähä & Primmer [43], 12 or 24 loci with pairwise *F*_ST_ values between hybridizing parental populations of 0.21 or 0.12, respectively, are required for the successful detection of F1 hybrids. In our case, all species pairs showed highly significant (*P*<0.001) genotypic differentiation (see also [34]). The highest *F*_ST_ value (only purebred individuals used) were recovered between *M. alcathoe* and *M. brandtii* (0.18). The lowest values were recorded between *M. mystacinus* and two other species: *M. alcathoe* (0.14) and *M. brandtii* (0.13). In most cases, the ‘absence’ of F1 hybrids in our data set could most probably be explained by a lack of power to detect them. However, the *F*_ST_ value between *M. alcathoe* and *M. brandtii* and the number of microsatellites used are close to that required in F1 analyses, and it cannot be excluded that the rarity of such animals in the wild is a result of assortative mating, i.e. the tendency for ‘like to mate with like’, shown by closely related species living in sympatry [48].

Species segregation in time (e.g., [24], [25], [49], [50]) and space [25] during the swarming season may also have some influence on the number of hybrids. In the Polish Carpathians, *M. brandtii* shows peak activity at the onset of swarming (July and at the beginning of August), *M. mystacinus* is active throughout the entire swarming period (July–September), whereas *M. alcathoe* swarms from the end of July to the middle of September. Swarming activity also occurs earlier at high elevation, where *M. mystacinus* is more frequent, than at lower elevations, where *M. brandtii* and *M. alcathoe* are more often encountered ([17], [25], authors' unpubl. data). In such a case, one would expect fewer hybrids between *M. brandtii* and the other two taxa as a result of this difference in time of activity, but also fewer hybrids between *M. brandtii* and *M. alcathoe* versus *M. mystacinus* due to spacial segregation. Nevertheless, the hybridization rate in *M. alcathoe* appears to be much higher than in other two species, and this may be related to the relatively low density of the former species in most swarming sites we studied (e.g., [17], [25]). In this situation, interspecific mating is probably facilitated by the asymmetry in the abundance of hybridizing taxa (e.g., [6]).

A further factor to be taken into account is that in terms of phylogenetic relationships, *M. brandtii* is within a North American clade (e.g., [51]), whereas *M. mystacinus* and *M. alcathoe* are more closely related to other Palaearctic species, and subsequently, to each other than either is to *M. brandtii* [32]. Such phylogenetic affinities may have important impact on the hybridization success, and indeed, based on the STRUCTURE results, relatively more hybridization is detected between *M. mystacinus* and *M. alcathoe* than between *M. brandtii* and the other species (Figure 3A). Assuming that the likelihood of producing hybrids is approximately the same in all three species, based on the sample sizes for each species, *M. mystacinus* should have 2.69 hybrids with *M. alcathoe* and 7.65 hybrids with *M. brandtii*; in practice we had 15 and 7 hybrids, respectively.

On the other hand, it is remarkable that rather extensive hybridization in three species distributed sympatrically over large areas has not disrupted the integrity of their gene pools. Nevertheless, genetic divergence between the three species is high, suggesting that they have been separated for approximately 11–12.5 million years (Figure 4 in [52]). These values are well above the average time to hybrid inviability estimated for mammals, which is approximately 2–4 million years (reviewed by Fitzpatrick [53]).

There are striking differences in the ratio of males to females for *M. mystacinus*, *M. alcathoe*, and *M. brandtii* at the population level during swarming (1.6∶1, 1.7∶1, and 4∶1, respectively) and in individuals identified as hybrids (respectively 8∶1, 1.8∶1, and 4∶0 in STRUCTURE and 3.7∶1, 1∶1, and 16∶1 in NEWHYBRIDS). It appears that the bias is stronger in hybrids than in the general population (although not for all species), but the observed differences are not statistically significant (in all cases *P*>0.05; two-tailed difference test between two proportions available in Statistica 64 ver. 10, StatSoft, Inc.). On the other hand, based on Haldane's rule, it is the heterogametic sex that is likely to be absent, rare or sterile in interspecific hybrids [54]. In *M. brandtii* we observed several backcrosses, and also a small fraction of F2 hybrids. Perhaps, as already proposed by others (e.g., [55]), Haldane's rule is effective for sterility but relatively weak for inviability.

Another explanation could be that the observed variation was caused by migrants from adjacent populations with divergent gene frequencies. However, this explanation is unlikely given the presence of well-mixed assemblages and the relatively low level of genetic differentiation between swarming sites [34].

No general rule about morphological features of hybrid individuals among our three study species (except for male *M. brandtii*) can be deduced from our research. Hybrids with parental morphology, intermediate morphology or phenotype skewed toward one of parents have been reported, but this is not surprising given the wide range of genomic variation detected.

In our opinion, there are several factors facilitating hybridization among these species: i) swarming behaviour at swarming sites, where high numbers of bats belonging to several species meet; ii) male-biased sex ratio during swarming period; and iii) the fact that all these bats belong to a group of polygynous species. Males of such species might be keen to mate with every potential female because they have low investment costs per mating relative to females. However, in seasonally breeding species, such as temperate bats, testes recrudesce, and it cannot be excluded that they would only do this if sperm production is costly in some way. Furthermore, in many bats mating occurs after testes have regressed so males cannot replenish their sperm supplies [56]. In such a case they should allocate ejaculates prudently, but all that is needed for polygyny (or even promiscuity) is to have larger ejaculate storage for more matings. In fact, mating in these taxa is not random. Bogdanowicz *et al.* [34] showed that during the swarming period, 4.1%, 5.9%, and 8.7% of individuals of *M. mystacinus*, *M. brandtii*, and *M. alcathoe* respectively were full siblings. A much higher percentage of individuals (47.0–47.8%) was assigned to kin groups representing bats sharing one parent (i.e., half siblings). These values suggest the possibility of females mating selectively with the same male in more than one year and indicate that reproductive success in these bats may be skewed towards some males or male lineages as in swarming *Myotis lucifugus* [57]. This would further reduce the number of available females for intraspecific matings and may serve to ‘force’ a higher frequency of interspecific matings. In contrast, during winter there is no such mechanism of female mate selection operating because most bats hibernate (and, without going into monthly details, sex ratio is around 1∶1), and any active male has free access to any female of its own species.

The presence of hybrids indicates some degree of randomness in mate selection, what may be profitable in evolutionary terms, facilitating invading species to colonize new areas, with potentially important consequences in evolutionary biology, speciation, biodiversity, and conservation. These species hybridize more frequently than would generally be expected, and some of their hybrids appear to be sufficiently fertile to backcross to the parents. At swarming sites at three caves in the Carpathian Mountains, we recorded 19 of the 25 bat species found in Poland [25], including at least 7 species known to produce hybrids (*Myotis myotis* and *M. oxygnathus* [7], [8], *Eptesicus nilssonii* and *E. serotinus* [12], and the three species examined herein). Such swarming sites certainly represent unique chiropteran hybrid hotspots, which facilitate gene exchange, may play an important role in speciation, and should be treated as focal points for the conservation of biodiversity and evolutionary processes [58]. In any case, in the Polish Carpathians alone there are 2,000–2,100 caves within the distribution range of our three study species, and most of them are used by bats as swarming sites [17], [25]. Hybrids (as defined by STRUCTURE, *Tq*<0.90) were discovered in ca. 52% (14 out of 27) caves we studied.

These figures indicate that hybridization hotspots in swarming bats may be extremely common, facilitating the exchange of genetic material at a scale not seen in any other species of mammal. As there is a high amount of cryptic diversity among European bats (e.g., [35], [59], [60]), with a good potential to hybridize (which can even lead to generation of new mammalian lineages with the species characteristics [11]), the role of swarming sites in maintaining this high level may be more important than previously thought.

## Supporting Information

Appendix S1 **Microsatellite genotypes.** The ‘ID’ column is an individual identifier. Subsequent columns provide species abreviations (Sp.) and genotypes for the 14 autosomal microsatellite loci as nominal sizes in base pairs. Mys – *M. mystacinus*, Bra – *M. brandtii*, Alc – *M. alcathoe*. Please note that the gender has been noted in all captured bats but this information was not available for some genetic samples.
(DOC)
