# Supplementary material for: Hybridization Hotspots at Bat Swarming Sites
Source: PLoS One. 2012 Dec 28;7(12):e53334. doi: 10.1371/journal.pone.0053334 (PMC3532499; doi:10.1371/journal.pone.0053334)
Supplement: Appendix S1 — Microsatellite genotypes. The ‘ID’ column is an individual identifier. Subsequent columns provide species abreviations (Sp.) and genotypes for the 14 autosomal microsatellite loci as nominal sizes in base pairs. Mys – M. mystacinus, Bra – M. brandtii, Alc – M. alcathoe. Please note that the gender has been noted in all captured bats but this information was not available for some genetic samples. (DOC) [file pone.0053334.s001.doc]

**Supplementary information**

**Appendix S1**. Microsatellite genotypes. The ‘ID’ column is an individual identifier. Subsequent columns provide species abreviations (Sp.) and genotypes for the 14 autosomal microsatellite loci as nominal sizes in base pairs. Mys – *M. mystacinus*, Bra – *M. brandtii*, Alc – *M. alcathoe.* Please note that the gender has been noted in all captured bats but this information was not available for some genetic samples.

| ID | Sp. | D9 | | D15 | | F19 | | G30 | | H29 | | EF15 Mluc | | G2 Mluc | | G31 Mluc | | G6 Mluc | | B8-Mluc | | D15-Mluc | | F19-Mluc | | G30-Mluc | | H23-Mluc | |
| --- | --- | --- | --- | --- | --- | --- | --- | --- | --- | --- | --- | --- | --- | --- | --- | --- | --- | --- | --- | --- | --- | --- | --- | --- | --- | --- | --- | --- | --- |
| WK_MJ4483 | Mys | 138 | 156 | 86 | 94 | 197 | 219 | 131 | 135 | 168 | 170 | - | - | 345 | 345 | 203 | 203 | 128 | 128 | 416 | 416 | 334 | 342 | 407 | 445 | 291 | 295 | 148 | 162 |
| WK_FJ5218 | Mys | 122 | 138 | 100 | 100 | 193 | 199 | 141 | 147 | 170 | 172 | 225 | 229 | 345 | 345 | 203 | 211 | 110 | 140 | 390 | 404 | 348 | 348 | 407 | 421 | 301 | 307 | 154 | 158 |
| WK_FJ5217 | Mys | 138 | 144 | 76 | 100 | 207 | 209 | 119 | 137 | 170 | 170 | 215 | 219 | 343 | 345 | 209 | 211 | 126 | 128 | 416 | 418 | 324 | 348 | 433 | 435 | 279 | 297 | 152 | 154 |
| WK_FA4865 | Mys | 134 | 144 | 70 | 100 | 207 | 207 | 141 | 147 | 170 | 170 | 225 | 225 | 345 | 345 | 205 | 211 | 140 | 142 | 404 | 408 | 318 | 348 | 435 | 435 | 301 | 307 | 136 | 152 |
| WK_FA4864 | Mys | 134 | 142 | 92 | 92 | 193 | 209 | 129 | 141 | 170 | 176 | 217 | 217 | 345 | 345 | 203 | 211 | 108 | 128 | 406 | 430 | 340 | 340 | 421 | 437 | 289 | 301 | 160 | 162 |
| WK_FA4863 | Mys | 136 | 138 | 86 | 124 | 209 | 219 | 129 | 137 | 170 | 170 | 213 | 221 | 345 | 347 | 197 | 207 | 108 | 140 | 400 | 400 | 334 | 370 | 437 | 447 | 289 | 297 | 156 | 158 |
| WK_FA4862 | Mys | 134 | 138 | 92 | 92 | 207 | 213 | 121 | 147 | 170 | 176 | 215 | 223 | 343 | 345 | 203 | 211 | 108 | 128 | 410 | 430 | 340 | 340 | 435 | 439 | 281 | 307 | 160 | 160 |
| WK_FA4861 | Mys | 134 | 142 | 72 | 100 | 207 | 207 | 129 | 137 | 168 | 170 | 215 | 219 | 345 | 345 | 201 | 211 | 108 | 128 | 398 | 406 | 318 | 348 | 435 | 435 | 289 | 297 | 136 | 136 |
| WK_FA4860 | Mys | 126 | 134 | 86 | 92 | 207 | 221 | 147 | 149 | 168 | 184 | 217 | 219 | 345 | 345 | 207 | 213 | 128 | 136 | 396 | 410 | 334 | 338 | 435 | 447 | 307 | 307 | 154 | 160 |
| WK_FA4859 | Mys | 132 | 150 | 86 | 88 | 211 | 225 | 137 | 141 | 170 | 170 | 219 | 229 | 343 | 343 | 203 | 211 | 126 | 128 | 404 | 410 | 334 | 336 | 441 | 453 | 297 | 301 | 136 | 158 |
| WK_FA4858 | Mys | 134 | 136 | 72 | 82 | 207 | 217 | 119 | 121 | 170 | 170 | 227 | 227 | 343 | 345 | 205 | 209 | 126 | 126 | 402 | 434 | 318 | 340 | 435 | 445 | 279 | 281 | 158 | 160 |
| WK_F04856 | Mys | 142 | 146 | 72 | 94 | 205 | 217 | 129 | 135 | 170 | 174 | 221 | 227 | 343 | 345 | 203 | 209 | 128 | 128 | 410 | 412 | 318 | 342 | 433 | 445 | 289 | 295 | 154 | 158 |
| TR_MJ4427 | Mys | 128 | 130 | 92 | 98 | 199 | 213 | 145 | 151 | 170 | 170 | 213 | 213 | 345 | 345 | 211 | 211 | 128 | 128 | 408 | 420 | 340 | 346 | 407 | 439 | 305 | 311 | 154 | 158 |
| TR_MJ4426 | Mys | 130 | 134 | 86 | 92 | 193 | 193 | 121 | 121 | 168 | 170 | 215 | 223 | 345 | 345 | 205 | 215 | 108 | 142 | 404 | 418 | 334 | 340 | 421 | 421 | 281 | 281 | 136 | 158 |
| TR_MA4902 | Mys | 138 | 140 | 98 | 98 | 193 | 229 | 133 | 145 | 180 | 180 | 217 | 223 | 345 | 345 | 209 | 211 | 126 | 128 | 396 | 404 | 346 | 346 | 421 | 455 | 293 | 305 | 152 | 154 |
| TR_MA4897 | Mys | 122 | 140 | 86 | 100 | 201 | 207 | 119 | 129 | 170 | 178 | 219 | 221 | 343 | 345 | 203 | 215 | 128 | 128 | 408 | 412 | 334 | 348 | 409 | 435 | 279 | 289 | 160 | 162 |
| TR_MA4429 | Mys | 132 | 140 | 98 | 98 | 211 | 211 | 129 | 153 | 164 | 170 | 213 | 217 | 343 | 345 | 211 | 211 | 126 | 138 | 404 | 428 | 346 | 346 | 435 | 437 | 289 | 313 | 136 | 154 |
| TR_M04857 | Mys | 130 | 130 | 92 | 94 | 207 | 221 | 129 | 149 | 170 | 170 | 209 | 227 | 343 | 345 | 187 | 211 | 128 | 138 | 406 | 408 | 340 | 342 | 435 | 449 | 289 | 309 | 136 | 162 |
| TR_M04671 | Mys | 120 | 140 | 92 | 96 | 207 | 213 | 141 | 143 | 170 | 178 | 219 | 229 | 343 | 345 | 209 | 211 | 128 | 132 | 410 | 426 | 340 | 344 | 423 | 435 | 303 | 311 | 152 | 162 |
| TR_M04669 | Mys | 138 | 138 | 86 | 92 | 195 | 199 | 119 | 121 | 170 | 172 | 215 | 221 | 345 | 345 | 207 | 211 | 126 | 128 | 384 | 434 | 334 | 340 | 409 | 423 | 279 | 281 | 158 | 158 |
| TR_M04668 | Mys | 140 | 156 | 86 | 92 | 207 | 207 | 119 | 131 | 170 | 182 | 213 | 223 | 345 | 345 | 203 | 211 | 110 | 110 | 406 | 410 | 334 | 340 | 435 | 435 | 279 | 291 | 152 | 160 |
| TR_M04667 | Mys | 138 | 138 | 92 | 98 | 211 | 219 | 133 | 141 | 170 | 170 | 215 | 219 | 345 | 345 | 207 | 211 | 128 | 136 | 390 | 400 | 338 | 346 | 437 | 445 | 293 | 301 | 160 | 162 |
| TR_M04663 | Mys | 124 | 126 | 86 | 92 | 207 | 211 | 129 | 135 | 170 | 170 | 227 | 227 | 343 | 345 | 209 | 213 | 112 | 128 | 416 | 420 | 334 | 340 | 421 | 435 | 289 | 295 | 154 | 156 |
| TR_M04659 | Mys | 122 | 130 | 92 | 98 | 197 | 215 | 119 | 121 | 166 | 172 | 201 | 201 | 343 | 345 | 203 | 207 | 138 | 138 | 388 | 408 | 340 | 346 | 407 | 441 | 279 | 281 | 156 | 160 |
| TR_M04655 | Mys | 118 | 128 | 76 | 100 | 193 | 207 | 117 | 143 | 168 | 170 | - | - | 343 | 345 | 211 | 213 | 126 | 138 | 400 | 414 | 324 | 348 | 421 | 435 | 277 | 303 | 134 | 158 |
| TR_M04654 | Mys | 120 | 130 | 92 | 92 | 193 | 199 | 129 | 143 | 170 | 184 | 221 | 223 | 345 | 347 | 213 | 215 | 110 | 128 | 396 | 410 | 318 | 340 | 409 | 421 | 289 | 303 | 146 | 160 |
| TR_M04653 | Mys | 138 | 140 | 92 | 96 | 193 | 207 | 119 | 129 | 168 | 170 | 215 | 231 | 343 | 345 | 205 | 213 | 128 | 128 | 384 | 398 | 340 | 344 | 421 | 435 | 279 | 289 | 156 | 158 |
| TR_M04652 | Mys | 132 | 140 | 86 | 92 | 193 | 203 | 119 | 121 | 168 | 170 | 215 | 217 | 345 | 345 | 207 | 207 | 136 | 140 | 410 | 432 | 334 | 340 | 421 | 431 | 279 | 281 | 160 | 160 |
| TR_M04644 | Mys | 130 | 130 | 92 | 92 | 197 | 207 | 141 | 149 | 170 | 180 | 213 | 215 | 345 | 345 | 203 | 215 | 110 | 126 | 384 | 414 | 340 | 340 | 407 | 435 | 301 | 309 | 136 | 154 |
| TR_M04642 | Mys | 138 | 162 | 92 | 118 | 193 | 193 | 119 | 133 | 170 | 172 | 209 | 217 | 345 | 345 | 203 | 211 | 128 | 142 | 410 | 418 | 340 | 364 | 421 | 421 | 279 | 293 | 158 | 162 |
| TR_M04640 | Mys | 118 | 122 | 92 | 94 | 193 | 209 | 149 | 149 | 170 | 170 | 225 | 227 | 345 | 345 | 207 | 213 | 140 | 140 | 410 | 432 | 340 | 342 | 421 | 435 | 309 | 309 | 136 | 158 |
| TR_M01443 | Mys | 122 | 130 | 92 | 92 | 213 | 217 | 129 | 129 | 170 | 170 | 215 | 227 | 345 | 345 | 201 | 211 | 110 | 112 | 400 | 404 | 340 | 340 | 441 | 441 | 289 | 289 | 152 | 158 |
| TR_FJ4428 | Mys | 142 | 142 | 72 | 92 | 221 | 227 | 141 | 141 | 164 | 170 | 223 | 223 | 343 | 345 | 211 | 211 | 126 | 138 | 404 | 404 | 318 | 340 | 447 | 453 | 289 | 301 | 154 | 160 |
| TR_FJ4425 | Mys | 122 | 134 | 94 | 94 | 193 | 197 | 119 | 119 | 168 | 170 | 217 | 229 | 345 | 345 | 203 | 211 | 126 | 138 | 400 | 414 | 340 | 342 | 407 | 421 | 279 | 279 | 140 | 152 |
| TR_FA4901 | Mys | 140 | 144 | 72 | 98 | 217 | 217 | 119 | 147 | 170 | 170 | 217 | 217 | 345 | 345 | 205 | 207 | 112 | 140 | 396 | 402 | 318 | 346 | 443 | 443 | 279 | 307 | 134 | 160 |
| TR_FA4430 | Mys | 122 | 140 | 88 | 94 | 193 | 199 | 139 | 151 | 180 | 188 | 225 | 225 | 343 | 345 | 203 | 215 | 126 | 128 | 406 | 412 | 334 | 342 | 409 | 421 | 299 | 311 | 158 | 160 |
| TR_F04670 | Mys | 120 | 140 | 98 | 98 | 207 | 209 | 119 | 121 | 168 | 180 | 213 | 215 | 345 | 345 | 201 | 211 | 110 | 136 | 408 | 432 | 346 | 346 | 435 | 437 | 279 | 281 | 136 | 150 |
| TR_F04666 | Mys | 126 | 140 | 92 | 94 | 199 | 205 | 129 | 147 | 170 | 170 | 223 | 229 | 343 | 345 | 209 | 211 | 126 | 128 | 384 | 414 | 340 | 342 | 409 | 433 | 289 | 307 | 136 | 162 |
| TR_F04664 | Mys | 122 | 122 | 92 | 92 | 197 | 207 | 127 | 141 | 170 | 170 | 217 | 217 | 343 | 345 | 207 | 213 | 108 | 136 | 408 | 426 | 340 | 340 | 407 | 435 | 287 | 301 | 142 | 156 |
| TR_F04661 | Mys | 142 | 142 | 72 | 102 | 197 | 207 | 141 | 141 | 170 | 170 | 215 | 225 | 345 | 345 | 207 | 213 | 110 | 128 | 408 | 410 | 318 | 350 | 407 | 435 | 289 | 301 | 136 | 152 |
| TR_F04660 | Mys | 124 | 140 | 92 | 98 | 193 | 207 | 129 | 131 | 164 | 168 | 239 | 239 | 343 | 345 | 203 | 209 | 128 | 138 | 414 | 424 | 340 | 346 | 421 | 435 | 289 | 291 | 136 | 154 |
| TR_F04658 | Mys | 138 | 148 | 72 | 86 | 203 | 209 | 125 | 127 | 170 | 170 | 213 | 217 | 345 | 345 | 185 | 215 | 108 | 140 | 402 | 412 | 318 | 334 | 431 | 435 | 285 | 287 | 152 | 152 |
| TR_F04657 | Mys | 136 | 138 | 86 | 92 | 197 | 197 | 131 | 131 | 172 | 180 | 215 | 221 | 343 | 345 | 211 | 213 | 126 | 126 | 404 | 406 | 334 | 340 | 407 | 407 | 291 | 291 | 136 | 156 |
| TR_F04643 | Mys | 136 | 140 | 98 | 100 | 197 | 207 | 121 | 129 | 168 | 168 | 215 | 215 | 345 | 345 | 203 | 207 | 112 | 126 | 412 | 434 | 346 | 348 | 407 | 435 | 281 | 289 | 156 | 160 |
| TR_F04641 | Mys | 134 | 140 | 92 | 110 | 193 | 193 | 129 | 129 | 170 | 170 | 217 | 219 | 345 | 345 | 207 | 213 | 126 | 136 | 398 | 410 | 340 | 340 | 421 | 431 | 289 | 289 | 156 | 158 |
| TR_F04639 | Mys | 120 | 142 | 72 | 98 | 197 | 213 | 119 | 129 | 168 | 168 | 221 | 221 | 343 | 345 | 203 | 211 | 108 | 110 | 410 | 426 | 318 | 344 | 407 | 423 | 279 | 289 | 136 | 154 |
| TR_004678 | Mys | 144 | 152 | 92 | 100 | 193 | 197 | 119 | 145 | 168 | 168 | 213 | 229 | 343 | 345 | 187 | 209 | 108 | 136 | 388 | 404 | 340 | 348 | 407 | 421 | 279 | 305 | 152 | 160 |
| TR_004677 | Mys | 118 | 140 | 86 | 92 | 197 | 213 | 131 | 131 | 170 | 170 | 219 | 219 | 345 | 345 | 209 | 211 | 128 | 136 | 406 | 408 | 334 | 340 | 407 | 423 | 291 | 291 | 136 | 162 |
| TR_004482 | Mys | 142 | 142 | 72 | 88 | 193 | 193 | 135 | 151 | 170 | 184 | 219 | 225 | 345 | 345 | 203 | 209 | 140 | 140 | 400 | 400 | 318 | 336 | 421 | 421 | 295 | 311 | 136 | 146 |
| TR_004480 | Mys | 126 | 156 | 86 | 92 | 207 | 225 | 139 | 143 | 164 | 168 | 229 | 229 | 345 | 347 | 203 | 211 | 128 | 140 | 396 | 410 | 334 | 340 | 435 | 451 | 299 | 303 | 156 | 164 |
| TR_004479 | Mys | 122 | 132 | 92 | 98 | 193 | 197 | 121 | 121 | 168 | 168 | 215 | 217 | 343 | 345 | 213 | 213 | 110 | 140 | 406 | 408 | 340 | 346 | 407 | 421 | 281 | 287 | 154 | 154 |
| TR_004478 | Mys | 124 | 144 | 94 | 96 | 197 | 219 | 119 | 119 | 170 | 170 | 215 | 215 | 343 | 345 | 207 | 213 | 128 | 128 | 406 | 410 | 342 | 344 | 407 | 445 | 279 | 281 | 150 | 152 |
| TR_001546 | Mys | 130 | 140 | 92 | 98 | 193 | 193 | 129 | 149 | 166 | 170 | 215 | 225 | 345 | 345 | 201 | 207 | 128 | 142 | 410 | 428 | 340 | 346 | 421 | 445 | 289 | 289 | 152 | 156 |
| TR_001542 | Mys | 132 | 154 | 100 | 100 | 207 | 207 | 123 | 123 | - | - | 215 | 227 | 345 | 347 | 201 | 215 | 128 | 142 | 414 | 414 | 340 | 348 | 435 | 435 | 279 | 279 | 160 | 160 |
| TR_001541 | Mys | 138 | 138 | 92 | 96 | 199 | 203 | 129 | 143 | - | - | 217 | 229 | 343 | 343 | 207 | 215 | 102 | 108 | 384 | 414 | 340 | 344 | 409 | 431 | 289 | 289 | 154 | 154 |
| TR_001540 | Mys | 132 | 132 | 0 | 0 | 199 | 207 | 125 | 125 | 170 | 170 | 217 | 225 | 345 | 345 | 201 | 211 | 110 | 140 | 432 | 432 | 368 | 368 | 407 | 407 | 285 | 301 | 136 | 146 |
| TR_001539 | Mys | 126 | 132 | 88 | 92 | 197 | 225 | 121 | 135 | - | - | 249 | 249 | 361 | 361 | 201 | 215 | 118 | 136 | 406 | 418 | 334 | 340 | 407 | 451 | 281 | 295 | 152 | 164 |
| TR_001529 | Mys | 118 | 136 | 94 | 100 | 203 | 217 | 139 | 139 | 170 | 170 | 213 | 219 | 345 | 345 | 211 | 211 | 128 | 128 | 406 | 410 | 342 | 348 | 431 | 443 | 299 | 299 | 154 | 162 |
| TR_001528 | Mys | 122 | 128 | 94 | 98 | 193 | 215 | 119 | 131 | 168 | 192 | 217 | 225 | 343 | 345 | 211 | 213 | 110 | 128 | 398 | 428 | 342 | 346 | 421 | 425 | 279 | 291 | 154 | 160 |
| TR_001527 | Mys | 150 | 152 | 100 | 100 | - | - | 129 | 147 | - | - | 215 | 217 | 345 | 345 | 207 | 211 | 110 | 142 | 400 | 402 | 326 | 348 | 407 | 407 | 289 | 307 | 160 | 160 |
| TA_MJ4440 | Mys | 132 | 142 | 92 | 92 | 193 | 193 | 143 | 145 | 168 | 170 | 217 | 217 | 345 | 347 | 201 | 213 | 110 | 136 | 396 | 426 | 340 | 340 | 421 | 421 | 303 | 305 | 136 | 154 |
| TA_MJ4423 | Mys | 132 | 132 | 92 | 98 | 199 | 199 | 119 | 141 | 170 | 182 | 215 | 221 | 323 | 325 | 181 | 187 | 108 | 116 | 398 | 406 | 340 | 346 | 409 | 427 | 279 | 301 | 152 | 152 |
| TA_MJ4422 | Mys | 138 | 140 | 78 | 92 | 215 | 221 | 129 | 151 | 170 | 178 | 211 | 231 | 345 | 345 | 211 | 215 | 108 | 112 | 412 | 426 | 326 | 340 | 441 | 447 | 289 | 311 | 154 | 156 |
| TA_MJ4411 | Mys | 132 | 142 | 92 | 98 | 197 | 197 | 119 | 147 | 168 | 170 | 217 | 217 | 345 | 345 | 201 | 211 | 128 | 140 | 406 | 408 | 340 | 346 | 407 | 407 | 279 | 307 | 156 | 158 |
| TA_MJ4310 | Mys | 126 | 150 | 92 | 92 | 205 | 213 | 131 | 151 | - | - | 213 | 233 | 345 | 345 | 205 | 207 | 126 | 142 | 404 | 436 | 340 | 340 | 433 | 439 | 291 | 311 | 152 | 160 |
| TA_MJ3545 | Mys | 128 | 130 | 86 | 98 | 193 | 217 | 129 | 145 | 168 | 184 | 219 | 219 | 345 | 345 | 209 | 209 | 128 | 140 | 410 | 412 | 334 | 346 | 421 | 443 | 289 | 303 | 154 | 158 |
| TA_MJ3542 | Mys | 140 | 140 | 86 | 92 | 197 | 205 | 129 | 145 | 170 | 184 | 215 | 215 | 345 | 345 | 205 | 211 | 126 | 138 | 400 | 412 | 334 | 340 | 407 | 433 | 289 | 305 | 136 | 156 |
| TA_MA4875 | Mys | 122 | 138 | 94 | 98 | 207 | 213 | 141 | 141 | 168 | 172 | 217 | 225 | 345 | 345 | 211 | 211 | 108 | 108 | 408 | 416 | 342 | 346 | 435 | 439 | 301 | 301 | 150 | 152 |
| TA_MA4874 | Mys | 122 | 128 | 92 | 98 | 193 | 209 | 139 | 141 | 168 | 182 | 215 | 215 | 345 | 345 | 207 | 209 | 128 | 138 | 402 | 410 | 340 | 346 | 421 | 437 | 299 | 301 | 152 | 156 |
| TA_MA4442 | Mys | 118 | 130 | 86 | 96 | 197 | 217 | 139 | 143 | - | - | 217 | 219 | 343 | 345 | 187 | 207 | 108 | 128 | 410 | 410 | 334 | 344 | 407 | 443 | 299 | 303 | 136 | 152 |
| TA_MA4439 | Mys | 138 | 148 | 92 | 100 | 213 | 217 | 121 | 131 | 168 | 170 | 215 | 215 | 345 | 345 | 201 | 203 | 126 | 138 | 406 | 414 | 338 | 348 | 423 | 443 | 281 | 291 | 136 | 158 |
| TA_MA4438 | Mys | 138 | 138 | 70 | 98 | 197 | 219 | 127 | 145 | 170 | 170 | 217 | 217 | 345 | 345 | 207 | 211 | 128 | 144 | 398 | 412 | 318 | 346 | 407 | 445 | 287 | 305 | 136 | 166 |
| TA_MA4420 | Mys | 136 | 152 | 92 | 92 | 199 | 217 | 143 | 151 | 168 | 178 | 217 | 219 | 345 | 345 | 203 | 207 | 128 | 128 | 410 | 434 | 338 | 340 | 407 | 443 | 303 | 311 | 154 | 156 |
| TA_MA4418 | Mys | 124 | 128 | 88 | 94 | 197 | 211 | 139 | 141 | 170 | 170 | 217 | 227 | 345 | 345 | 211 | 213 | 110 | 126 | 402 | 422 | 336 | 342 | 407 | 439 | 299 | 301 | 152 | 162 |
| TA_MA4417 | Mys | 136 | 152 | 86 | 98 | 197 | 197 | 121 | 127 | 170 | 174 | 217 | 217 | 345 | 347 | 205 | 209 | 110 | 110 | 412 | 412 | 334 | 346 | 407 | 407 | 281 | 287 | 136 | 164 |
| TA_MA4416 | Mys | 136 | 138 | 86 | 106 | 207 | 215 | 121 | 129 | 168 | 170 | 217 | 217 | 345 | 345 | 205 | 209 | 108 | 140 | 404 | 408 | 334 | 352 | 435 | 441 | 281 | 289 | 146 | 162 |
| TA_MA4415 | Mys | 134 | 134 | 92 | 92 | 207 | 207 | 119 | 121 | 168 | 170 | 217 | 217 | 345 | 345 | 205 | 213 | 128 | 128 | 400 | 410 | 340 | 340 | 435 | 435 | 279 | 281 | 136 | 160 |
| TA_MA4414 | Mys | 118 | 132 | 86 | 86 | 197 | 197 | 119 | 131 | 170 | 170 | 219 | 223 | 343 | 345 | 211 | 211 | 108 | 138 | 388 | 434 | 334 | 334 | 407 | 407 | 279 | 291 | 140 | 154 |
| TA_MA4413 | Mys | 128 | 136 | 86 | 92 | 199 | 207 | 129 | 135 | 174 | 176 | 215 | 225 | 343 | 343 | 203 | 205 | 126 | 126 | 412 | 424 | 334 | 340 | 409 | 435 | 289 | 295 | 136 | 162 |
| TA_MA4308 | Mys | 134 | 140 | 72 | 92 | 207 | 221 | 129 | 145 | 172 | 174 | 215 | 227 | 345 | 345 | 187 | 211 | 136 | 140 | 408 | 414 | 318 | 340 | 435 | 447 | 289 | 305 | 154 | 158 |
| TA_MA4307 | Mys | 122 | 142 | 92 | 94 | 213 | 219 | 131 | 151 | - | - | 215 | 217 | 345 | 347 | 205 | 215 | 128 | 138 | 384 | 404 | 338 | 342 | 441 | 447 | 291 | 311 | 138 | 156 |
| TA_MA4306 | Mys | 130 | 132 | 72 | 92 | 193 | 207 | 137 | 143 | - | - | 213 | 217 | 345 | 345 | 207 | 213 | 128 | 138 | 406 | 410 | 318 | 340 | 421 | 435 | 297 | 303 | 134 | 152 |
| TA_MA4305 | Mys | 122 | 130 | 72 | 72 | 193 | 207 | 119 | 129 | 170 | 170 | 213 | 213 | 343 | 345 | 203 | 209 | 110 | 136 | 396 | 412 | 318 | 318 | 421 | 435 | 279 | 289 | 136 | 160 |
| TA_MA3549 | Mys | 142 | 152 | 92 | 92 | 197 | 215 | 129 | 147 | 170 | 172 | 217 | 219 | 345 | 345 | 209 | 211 | 110 | 140 | 418 | 420 | 340 | 340 | 409 | 443 | 289 | 307 | 136 | 160 |
| TA_M04674 | Mys | 144 | 170 | 86 | 92 | 193 | 207 | 147 | 147 | 170 | 182 | 219 | 233 | 345 | 345 | 209 | 211 | 136 | 142 | 384 | 410 | 334 | 340 | 421 | 435 | 307 | 307 | 150 | 160 |
| TA_M04673 | Mys | 124 | 132 | 72 | 88 | 193 | 203 | 123 | 149 | 172 | 178 | 221 | 221 | 345 | 345 | 201 | 205 | 128 | 136 | 406 | 408 | 318 | 336 | 421 | 431 | 283 | 311 | 152 | 154 |
| TA_M04419 | Mys | 124 | 132 | 92 | 94 | 197 | 197 | 119 | 145 | 168 | 170 | 217 | 217 | 345 | 345 | 201 | 203 | 126 | 140 | 388 | 416 | 340 | 342 | 407 | 407 | 279 | 305 | 152 | 156 |
| TA_M04302 | Mys | 146 | 146 | 86 | 96 | 219 | 221 | 131 | 145 | 168 | 170 | 217 | 221 | 343 | 343 | 209 | 211 | 128 | 142 | 402 | 410 | 334 | 344 | 445 | 447 | 291 | 305 | 136 | 136 |
| TA_M04300 | Mys | 126 | 132 | 86 | 96 | 197 | 207 | 121 | 137 | 170 | 180 | 213 | 217 | 345 | 345 | 203 | 213 | 128 | 136 | 394 | 406 | 334 | 344 | 407 | 435 | 281 | 297 | 160 | 160 |
| TA_M04298 | Mys | 140 | 140 | 86 | 96 | 193 | 209 | 123 | 133 | 164 | 180 | 217 | 229 | 345 | 347 | 201 | 211 | 108 | 138 | 406 | 412 | 334 | 344 | 421 | 437 | 283 | 293 | 154 | 158 |
| TA_M04296 | Mys | 130 | 148 | 86 | 92 | 213 | 217 | 139 | 141 | 164 | 170 | 223 | 223 | 343 | 345 | 205 | 209 | 128 | 138 | 404 | 412 | 334 | 340 | 439 | 443 | 299 | 301 | 136 | 152 |
| TA_M04295 | Mys | 134 | 134 | 72 | 92 | 195 | 217 | 129 | 151 | 174 | 174 | 217 | 217 | 345 | 345 | 201 | 211 | 110 | 126 | 398 | 398 | 318 | 340 | 423 | 443 | 289 | 311 | 136 | 154 |
| TA_M04294 | Mys | 140 | 140 | 92 | 98 | 211 | 219 | 133 | 141 | 170 | 170 | 215 | 219 | 345 | 345 | 207 | 211 | 128 | 136 | 390 | 400 | 338 | 346 | 437 | 445 | 293 | 301 | 160 | 162 |
| TA_M04293 | Mys | 122 | 128 | 72 | 98 | 197 | 221 | 119 | 151 | 170 | 178 | 217 | 225 | 345 | 345 | 203 | 205 | 108 | 138 | - | - | - | - | - | - | - | - | - | - |
| TA_FJ4421 | Mys | 130 | 134 | 96 | 98 | 195 | 195 | 129 | 153 | 168 | 170 | 217 | 225 | 345 | 345 | 209 | 209 | 108 | 136 | 402 | 414 | 344 | 346 | 421 | 421 | 289 | 313 | 154 | 160 |
| TA_FJ4412 | Mys | 132 | 140 | 72 | 94 | 197 | 207 | 123 | 129 | 176 | 184 | 217 | 225 | 345 | 345 | 203 | 203 | 110 | 128 | 404 | 410 | 318 | 342 | 407 | 435 | 283 | 289 | 158 | 160 |
| TA_FJ4304 | Mys | 124 | 144 | 94 | 98 | 193 | 199 | 143 | 145 | 170 | 172 | 217 | 227 | 345 | 345 | 209 | 213 | 128 | 138 | 404 | 404 | 342 | 346 | 409 | 421 | 289 | 305 | 154 | 160 |
| TA_FJ3547 | Mys | 118 | 136 | 92 | 92 | 205 | 207 | 133 | 147 | 170 | 170 | 225 | 225 | 343 | 345 | 205 | 205 | 126 | 142 | 412 | 412 | 340 | 340 | 433 | 435 | 293 | 307 | 152 | 160 |
| TA_FJ3535 | Mys | 130 | 142 | 92 | 98 | 197 | 221 | 129 | 141 | 170 | 170 | 215 | 217 | 345 | 345 | 205 | 205 | 126 | 128 | 396 | 402 | 340 | 346 | 407 | 447 | 289 | 301 | 136 | 136 |
| TA_FJ3534 | Mys | 124 | 146 | 92 | 92 | 197 | 207 | 121 | 129 | 168 | 170 | 217 | 225 | 343 | 347 | 201 | 205 | 108 | 138 | 398 | 424 | 318 | 340 | 407 | 435 | 281 | 289 | 156 | 156 |
| TA_FJ3532 | Mys | 130 | 148 | 92 | 92 | 193 | 195 | 121 | 147 | 168 | 170 | 221 | 223 | 345 | 345 | 207 | 207 | 112 | 128 | 402 | 420 | 318 | 340 | 421 | 423 | 281 | 307 | 148 | 154 |
| TA_FA4441 | Mys | 126 | 132 | 86 | 92 | 207 | 217 | 141 | 143 | 170 | 170 | 231 | 231 | 343 | 345 | 211 | 211 | 126 | 140 | 396 | 442 | 334 | 342 | 435 | 443 | 301 | 303 | 152 | 158 |
| TA_FA4410 | Mys | 124 | 130 | 86 | 92 | 199 | 217 | 129 | 141 | 168 | 170 | 217 | 223 | 343 | 345 | 205 | 211 | 126 | 128 | 408 | 410 | 334 | 340 | 407 | 443 | 289 | 301 | 136 | 162 |
| TA_FA4309 | Mys | 130 | 130 | 76 | 92 | 193 | 207 | 121 | 121 | 168 | 170 | 227 | 229 | 343 | 345 | 201 | 203 | 108 | 128 | 396 | 410 | 324 | 340 | 421 | 435 | 281 | 289 | 140 | 154 |
| TA_FA4303 | Mys | 132 | 132 | 94 | 98 | 197 | 221 | 119 | 129 | 168 | 170 | 215 | 225 | 345 | 345 | 205 | 207 | 128 | 128 | 404 | 406 | 342 | 346 | 407 | 447 | 279 | 289 | 136 | 154 |
| TA_FA3548 | Mys | 138 | 144 | 86 | 94 | 199 | 207 | 129 | 135 | - | - | 225 | 231 | 345 | 345 | 203 | 211 | 128 | 138 | 402 | 438 | 334 | 342 | 409 | 435 | 289 | 295 | 136 | 152 |
| TA_FA3546 | Mys | 130 | 148 | 72 | 92 | 217 | 225 | 141 | 145 | 168 | 170 | 215 | 223 | 345 | 345 | 201 | 205 | 110 | 128 | 434 | 442 | 318 | 340 | 445 | 451 | 301 | 305 | 136 | 136 |
| TA_FA3543 | Mys | 138 | 142 | 78 | 86 | 217 | 225 | 119 | 145 | 170 | 170 | 217 | 223 | 343 | 345 | 201 | 209 | 112 | 138 | 412 | 416 | 326 | 334 | 443 | 451 | 279 | 305 | 156 | 158 |
| TA_FA3541 | Mys | 124 | 130 | 76 | 94 | 193 | 197 | 139 | 141 | 168 | 170 | - | - | 343 | 345 | 203 | 207 | 110 | 128 | 408 | 440 | 324 | 342 | 407 | 421 | 299 | 301 | 156 | 156 |
| TA_FA3540 | Mys | 126 | 136 | 92 | 94 | 195 | 217 | 119 | 135 | 168 | 176 | 215 | 229 | 345 | 345 | 201 | 215 | 108 | 128 | 410 | 438 | 340 | 340 | 423 | 443 | 279 | 295 | 156 | 158 |
| TA_FA3538 | Mys | 128 | 144 | 72 | 86 | 193 | 209 | 139 | 149 | 168 | 170 | 219 | 219 | 345 | 345 | 207 | 209 | 110 | 142 | 386 | 434 | 318 | 334 | 421 | 437 | 299 | 309 | 140 | 152 |
| TA_FA3537 | Mys | 136 | 140 | 92 | 94 | 207 | 227 | 129 | 135 | 170 | 170 | 225 | 225 | 345 | 345 | 211 | 211 | 126 | 138 | 398 | 406 | 338 | 342 | 435 | 453 | 289 | 295 | 160 | 160 |
| TA_FA3536 | Mys | 128 | 152 | 92 | 100 | 205 | 217 | 119 | 127 | 170 | 186 | 217 | 221 | 345 | 345 | 205 | 209 | 126 | 128 | 402 | 420 | 340 | 348 | 433 | 445 | 279 | 287 | 136 | 154 |
| TA_FA3533 | Mys | 140 | 140 | 92 | 98 | 197 | 197 | 123 | 123 | 172 | 178 | 217 | 217 | 345 | 345 | 207 | 211 | 108 | 128 | 402 | 414 | 340 | 346 | 407 | 407 | 281 | 281 | 142 | 154 |
| TA_FA3531 | Mys | 136 | 136 | 92 | 94 | 193 | 221 | 139 | 141 | 170 | 176 | 219 | 227 | 343 | 345 | 209 | 211 | 126 | 128 | 404 | 410 | 340 | 342 | 421 | 447 | 299 | 301 | 152 | 160 |
| TA_F04672 | Mys | 128 | 136 | 92 | 98 | 193 | 207 | 129 | 131 | 168 | 170 | 217 | 223 | 345 | 345 | 205 | 207 | 126 | 126 | 406 | 410 | 340 | 346 | 421 | 435 | 289 | 291 | 144 | 160 |
| TA_F04424 | Mys | 138 | 138 | 86 | 92 | 195 | 199 | 119 | 121 | 170 | 172 | 213 | 221 | 345 | 345 | 207 | 211 | 126 | 128 | 384 | 434 | 334 | 340 | 409 | 423 | 279 | 281 | 158 | 158 |
| TA_F04301 | Mys | 134 | 142 | 98 | 98 | 207 | 213 | 129 | 141 | 168 | 170 | 213 | 217 | 345 | 345 | 211 | 211 | 112 | 128 | 390 | 406 | 340 | 346 | 435 | 439 | 289 | 301 | 152 | 164 |
| TA_F04297 | Mys | 122 | 138 | 92 | 100 | 199 | 207 | 131 | 135 | 168 | 176 | 217 | 227 | 343 | 345 | 201 | 205 | 126 | 136 | 400 | 410 | 340 | 348 | 409 | 435 | 291 | 295 | 154 | 162 |
| TA_003544 | Mys | 130 | 144 | 72 | 98 | 193 | 205 | 133 | 141 | 170 | 176 | 219 | 229 | 345 | 345 | 203 | 205 | 108 | 128 | 384 | 402 | 318 | 346 | 421 | 433 | 293 | 301 | 154 | 156 |
| TA_003539 | Mys | 122 | 132 | 72 | 92 | 193 | 217 | 149 | 153 | 170 | 176 | 219 | 219 | 343 | 345 | 203 | 209 | 126 | 138 | 376 | 412 | 318 | 340 | 421 | 443 | 309 | 315 | 154 | 158 |
| TA_001545 | Mys | 122 | 132 | 92 | 92 | 199 | 199 | - | - | 174 | 174 | 225 | 225 | 343 | 345 | 205 | 211 | 126 | 136 | 404 | 440 | 340 | 340 | 409 | 409 | 287 | 301 | 160 | 160 |
| TA_001544 | Mys | 136 | 146 | 92 | 92 | 209 | 209 | 147 | 147 | - | - | 219 | 229 | 343 | 345 | 205 | 209 | 110 | 140 | 412 | 430 | 318 | 340 | 437 | 443 | 307 | 307 | 150 | 152 |
| TA_001543 | Mys | 138 | 138 | - | - | - | - | 147 | 149 | - | - | 217 | 229 | 345 | 345 | 203 | 213 | 128 | 128 | - | - | - | - | - | - | 305 | 305 | 162 | 162 |
| TA_001538 | Mys | 134 | 140 | 78 | 88 | 193 | 217 | 121 | 131 | 170 | 170 | 217 | 247 | 345 | 345 | 201 | 209 | 110 | 128 | - | - | - | - | 421 | 445 | 291 | 291 | 160 | 160 |
| TA_001537 | Mys | 118 | 142 | 98 | 98 | 197 | 205 | 119 | 129 | 170 | 172 | 249 | 249 | 359 | 359 | 201 | 209 | 118 | 118 | 410 | 410 | 334 | 334 | 407 | 433 | 279 | 279 | 156 | 156 |
| TA_001536 | Mys | 128 | 128 | 98 | 98 | 193 | 211 | 129 | 143 | 178 | 178 | 249 | 249 | 361 | 375 | 201 | 209 | 116 | 118 | 408 | 410 | 340 | 346 | 421 | 437 | 289 | 289 | 136 | 156 |
| TA_001535 | Mys | 134 | 166 | 88 | 92 | 197 | 199 | 119 | 153 | 170 | 174 | 217 | 225 | 345 | 345 | 201 | 215 | 112 | 140 | 400 | 400 | 336 | 338 | 407 | 409 | 279 | 279 | 146 | 152 |
| SB_M03386 | Mys | 132 | 160 | 92 | 100 | 193 | 217 | 121 | 153 | 180 | 186 | 217 | 225 | 343 | 345 | 211 | 213 | 110 | 128 | 400 | 406 | 340 | 348 | 421 | 443 | 281 | 313 | 152 | 152 |
| SB_M03385 | Mys | 132 | 140 | 94 | 96 | 191 | 197 | 135 | 147 | 170 | 170 | 219 | 219 | 343 | 345 | 205 | 211 | 128 | 140 | 376 | 422 | 342 | 344 | 407 | 419 | 295 | 307 | 146 | 154 |
| SB_M03384 | Mys | 130 | 142 | 98 | 100 | 197 | 197 | 119 | 145 | 168 | 170 | 219 | 219 | 345 | 345 | 205 | 207 | 110 | 126 | 410 | 412 | 346 | 348 | 407 | 431 | 279 | 303 | 152 | 152 |
| SB_M03382 | Mys | 136 | 152 | 98 | 98 | 199 | 199 | 129 | 131 | 170 | 182 | 217 | 227 | 345 | 345 | 209 | 209 | 110 | 140 | 410 | 414 | 346 | 346 | 409 | 409 | 289 | 291 | 156 | 160 |
| SB_M03381 | Mys | 122 | 132 | 92 | 100 | 207 | 227 | 119 | 119 | 170 | 170 | 215 | 227 | 345 | 345 | 201 | 211 | 110 | 112 | 402 | 402 | 340 | 348 | 435 | 453 | 279 | 279 | 136 | 140 |
| SB_F03387 | Mys | 122 | 142 | 92 | 92 | 205 | 223 | 121 | 123 | 170 | 184 | 215 | 219 | 345 | 345 | 207 | 211 | 110 | 144 | 404 | 412 | 340 | 340 | 433 | 449 | 281 | 283 | 136 | 160 |
| SB_F03383 | Mys | 130 | 142 | 86 | 100 | 193 | 215 | 119 | 147 | 170 | 172 | 219 | 229 | 343 | 345 | 203 | 213 | 126 | 128 | 430 | 438 | 334 | 348 | 421 | 441 | 279 | 307 | 154 | 156 |
| SB_FJ4999 | Mys | 138 | 144 | 92 | 100 | 193 | 217 | 121 | 131 | 164 | 170 | 219 | 227 | 345 | 347 | 201 | 203 | 128 | 140 | 390 | 392 | 340 | 348 | 421 | 443 | 281 | 291 | 158 | 160 |
| PS_M03378 | Mys | 128 | 140 | 92 | 98 | 195 | 199 | 119 | 145 | 168 | 170 | 249 | 249 | 361 | 361 | 199 | 199 | 122 | 124 | 416 | 418 | 340 | 346 | 409 | 423 | 279 | 305 | 142 | 162 |
| PS_M02868 | Mys | 126 | 142 | 96 | 100 | 193 | 197 | 127 | 141 | 176 | 182 | 229 | 229 | 343 | 345 | 201 | 213 | 110 | 128 | 390 | 404 | 344 | 348 | 407 | 421 | 287 | 301 | 154 | 156 |
| PS_M02866 | Mys | 122 | 134 | 92 | 98 | 197 | 199 | 129 | 141 | 170 | 176 | 217 | 225 | 345 | 345 | 201 | 207 | 126 | 138 | 402 | 408 | 338 | 346 | 407 | 409 | 289 | 301 | 152 | 156 |
| PS_F03377 | Mys | 138 | 138 | 86 | 92 | 193 | 207 | 129 | 151 | 170 | 180 | 217 | 219 | 343 | 347 | 209 | 213 | 126 | 128 | 410 | 414 | 334 | 340 | 421 | 435 | 289 | 311 | 146 | 152 |
| PS_F03376 | Mys | 126 | 138 | 92 | 100 | 193 | 199 | 121 | 123 | 168 | 170 | 215 | 225 | 345 | 345 | 201 | 213 | 112 | 138 | 412 | 432 | 340 | 348 | 409 | 421 | 281 | 283 | 142 | 144 |
| PS_F02643 | Mys | 138 | 142 | 72 | 98 | 199 | 217 | 125 | 127 | 170 | 174 | 217 | 229 | 343 | 345 | 205 | 209 | 110 | 140 | 406 | 412 | 318 | 346 | 409 | 443 | 285 | 287 | 156 | 160 |
| PS_F02641 | Mys | 130 | 140 | 98 | 98 | 193 | 207 | 119 | 121 | 168 | 170 | 213 | 221 | 345 | 345 | 201 | 207 | 126 | 126 | 408 | 410 | 346 | 346 | 421 | 435 | 279 | 281 | 134 | 160 |
| PS_M02647 | Mys | 136 | 140 | 100 | 100 | 213 | 223 | 121 | 137 | 168 | 172 | 215 | 227 | 345 | 345 | 211 | 211 | 110 | 126 | 388 | 416 | 348 | 348 | 439 | 449 | 281 | 297 | 160 | 160 |
| PS_M02646 | Mys | 128 | 160 | 86 | 98 | 193 | 199 | 119 | 119 | 170 | 174 | 219 | 225 | 345 | 345 | 207 | 211 | 126 | 126 | 398 | 414 | 334 | 346 | 409 | 421 | 279 | 279 | 158 | 162 |
| PS_M02642 | Mys | 128 | 138 | 88 | 98 | 193 | 225 | 123 | 145 | 174 | 178 | 219 | 219 | 343 | 343 | 203 | 203 | 128 | 144 | 408 | 408 | 336 | 346 | 421 | 451 | 283 | 305 | 160 | 160 |
| GO_MA4955 | Mys | 128 | 136 | 92 | 94 | 197 | 223 | 121 | 147 | 168 | 170 | 225 | 225 | 345 | 345 | 203 | 205 | 110 | 126 | 408 | 410 | 338 | 342 | 407 | 449 | 281 | 307 | 152 | 164 |
| GO_MA4954 | Mys | 136 | 140 | 94 | 100 | 197 | 197 | 129 | 147 | 168 | 178 | 219 | 231 | 345 | 345 | 203 | 211 | 128 | 128 | 398 | 420 | 342 | 348 | 407 | 431 | 289 | 307 | 152 | 162 |
| GO_MA4950 | Mys | 142 | 148 | 72 | 96 | 197 | 197 | 141 | 153 | 168 | 170 | 219 | 219 | 345 | 345 | 203 | 203 | 110 | 110 | 406 | 406 | 318 | 344 | 407 | 421 | 301 | 313 | 156 | 158 |
| GO_MA4941 | Mys | 128 | 154 | 92 | 98 | 207 | 229 | 119 | 129 | 168 | 184 | 215 | 219 | 343 | 345 | 211 | 221 | 110 | 128 | 410 | 424 | 340 | 346 | 435 | 455 | 279 | 289 | 154 | 158 |
| GO_MA4936 | Mys | 132 | 140 | 72 | 92 | 199 | 207 | 121 | 131 | 170 | 182 | 217 | 219 | 345 | 347 | 187 | 209 | 110 | 146 | 404 | 412 | 318 | 340 | 409 | 435 | 279 | 289 | 152 | 156 |
| GO_MA4934 | Mys | 132 | 140 | 92 | 96 | 193 | 217 | 119 | 131 | 168 | 174 | 215 | 219 | 345 | 345 | 203 | 203 | 110 | 138 | 406 | 446 | 340 | 344 | 421 | 443 | 279 | 291 | 154 | 156 |
| GO_FJ4953 | Mys | 134 | 152 | 94 | 98 | 197 | 197 | 121 | 139 | 168 | 170 | 215 | 215 | 345 | 345 | 207 | 211 | 128 | 128 | 400 | 434 | 342 | 346 | 407 | 407 | 281 | 299 | 136 | 148 |
| GO_FJ4937 | Mys | 134 | 134 | 78 | 98 | 197 | 205 | 141 | 151 | 168 | 170 | 215 | 225 | 345 | 345 | 201 | 213 | 138 | 138 | 384 | 390 | 324 | 346 | 407 | 433 | 301 | 311 | 142 | 152 |
| GO_FA4951 | Mys | 126 | 144 | 94 | 100 | 193 | 193 | 119 | 143 | 170 | 178 | 217 | 217 | 345 | 345 | 201 | 211 | 110 | 128 | 414 | 442 | 342 | 348 | 421 | 421 | 279 | 303 | 160 | 162 |
| GO_FA4939 | Mys | 122 | 140 | 92 | 96 | 215 | 219 | 139 | 141 | 170 | 170 | 219 | 227 | 345 | 347 | 201 | 209 | 110 | 128 | 384 | 406 | 340 | 344 | 441 | 445 | 299 | 301 | 158 | 160 |
| GO_FA4938 | Mys | 124 | 128 | 92 | 94 | 207 | 227 | 119 | 121 | 168 | 168 | 219 | 219 | 345 | 345 | 211 | 217 | 110 | 140 | 406 | 408 | 340 | 342 | 435 | 453 | 279 | 281 | 154 | 158 |
| BW_FA4882 | Mys | 138 | 142 | 92 | 96 | 199 | 211 | 119 | 129 | 170 | 184 | 217 | 223 | 345 | 345 | 207 | 211 | 126 | 136 | 402 | 412 | 340 | 344 | 409 | 437 | 279 | 289 | 154 | 156 |
| BW_MJ4448 | Mys | 134 | 156 | 72 | 98 | 187 | 207 | 141 | 151 | 168 | 176 | 217 | 219 | 345 | 345 | 199 | 203 | 126 | 128 | 394 | 422 | 318 | 346 | 397 | 435 | 301 | 311 | 154 | 160 |
| BW_MA4883 | Mys | 132 | 138 | 86 | 92 | 193 | 207 | 121 | 143 | 164 | 170 | 219 | 227 | 343 | 345 | 201 | 203 | 108 | 128 | 394 | 404 | 334 | 340 | 421 | 435 | 281 | 303 | 140 | 140 |
| BW_MA4866 | Mys | 132 | 142 | 70 | 92 | 195 | 207 | 119 | 139 | 170 | 170 | 217 | 223 | 345 | 345 | 187 | 203 | 126 | 128 | 402 | 434 | 318 | 340 | 423 | 435 | 279 | 299 | 158 | 160 |
| BW_MA4292 | Mys | 120 | 136 | 98 | 98 | 197 | 211 | 129 | 129 | 168 | 170 | 207 | 231 | 343 | 345 | 211 | 213 | 108 | 128 | 396 | 410 | 346 | 346 | 407 | 437 | 289 | 289 | 152 | 160 |
| BW_FJ4450 | Mys | 136 | 136 | 92 | 92 | 203 | 219 | 131 | 151 | 168 | 180 | 213 | 217 | 345 | 345 | 201 | 207 | 126 | 128 | 412 | 414 | 340 | 340 | 431 | 445 | 291 | 311 | 150 | 166 |
| BW_FJ4447 | Mys | 120 | 150 | 92 | 98 | 193 | 195 | 131 | 143 | 170 | 184 | 215 | 229 | 345 | 345 | 203 | 205 | 112 | 126 | 396 | 404 | 340 | 346 | 421 | 423 | 291 | 303 | 136 | 154 |
| BW_FJ4443 | Mys | 126 | 140 | 86 | 96 | 207 | 213 | 121 | 129 | 168 | 170 | 219 | 223 | 345 | 345 | 203 | 207 | 110 | 128 | 410 | 418 | 334 | 344 | 435 | 439 | 281 | 289 | 136 | 156 |
| PI_MJ4465 | Mys | 132 | 142 | - | - | 207 | 207 | - | - | 184 | 184 | - | - | 345 | 345 | 203 | 207 | 108 | 126 | 404 | 414 | 348 | 348 | 421 | 435 | 289 | 305 | 154 | 162 |
| PI_MJ4464 | Mys | 162 | 162 | 72 | 92 | 207 | 221 | 119 | 135 | 168 | 176 | 227 | 229 | 345 | 345 | 187 | 203 | 112 | 140 | 398 | 398 | 318 | 340 | 435 | 447 | 279 | 295 | 146 | 150 |
| PI_M04679 | Mys | 140 | 148 | 92 | 98 | 197 | 221 | 129 | 145 | 170 | 174 | 217 | 217 | 343 | 345 | 203 | 209 | 128 | 128 | 400 | 406 | 340 | 346 | 407 | 449 | 289 | 305 | 136 | 152 |
| PI_M04656 | Mys | 140 | 144 | 72 | 96 | 203 | 207 | 131 | 145 | 168 | 170 | 219 | 221 | 343 | 345 | 203 | 203 | 128 | 138 | 406 | 412 | 318 | 340 | 431 | 435 | 291 | 305 | 136 | 158 |
| PI_M04648 | Mys | 122 | 138 | 72 | 116 | 199 | 209 | 125 | 143 | 168 | 168 | 227 | 227 | 343 | 345 | 209 | 209 | 110 | 128 | 394 | 416 | 318 | 362 | 409 | 437 | 285 | 303 | 158 | 160 |
| PI_M04647 | Mys | 140 | 142 | 92 | 92 | 203 | 207 | 129 | 149 | 170 | 170 | 217 | 217 | 345 | 345 | 207 | 215 | 110 | 128 | 404 | 410 | 340 | 340 | 431 | 435 | 289 | 311 | 152 | 156 |
| BS_MA4912 | Mys | 130 | 138 | 86 | 100 | 207 | 213 | 119 | 147 | 168 | 178 | 213 | 219 | 345 | 347 | 207 | 211 | 108 | 126 | 396 | 414 | 334 | 348 | 435 | 439 | 279 | 307 | 140 | 160 |
| BS_MA2468 | Mys | 138 | 138 | 94 | 96 | 197 | 197 | 121 | 139 | 170 | 170 | 217 | 243 | 341 | 341 | 205 | 211 | 102 | 106 | 430 | 446 | 342 | 344 | 407 | 407 | 281 | 299 | 136 | 156 |
| BS_MA2466 | Mys | 124 | 156 | 78 | 86 | 193 | 207 | 121 | 131 | 168 | 178 | 221 | 227 | 343 | 345 | 203 | 211 | 128 | 130 | 400 | 402 | 324 | 334 | 421 | 435 | 281 | 291 | 156 | 156 |
| BS_MA2465 | Mys | 122 | 138 | 92 | 98 | 193 | 209 | 139 | 143 | 168 | 170 | 219 | 219 | 341 | 341 | 207 | 211 | 100 | 102 | 412 | 422 | 340 | 346 | 421 | 437 | 299 | 303 | 152 | 160 |
| BS_MA2463 | Mys | 144 | 164 | 92 | 92 | 205 | 217 | 129 | 151 | 164 | 170 | 221 | 227 | 343 | 345 | 203 | 211 | 128 | 130 | 404 | 410 | 340 | 340 | 433 | 445 | 289 | 311 | 158 | 162 |
| BA_MJ4408 | Mys | 136 | 152 | 88 | 88 | 193 | 207 | 119 | 149 | 168 | 170 | 219 | 219 | 345 | 345 | 203 | 211 | 108 | 108 | 412 | 436 | 334 | 334 | 421 | 435 | 279 | 309 | 146 | 160 |
| BA_MJ4406 | Mys | 124 | 128 | 88 | 98 | 193 | 223 | 139 | 141 | 170 | 182 | 223 | 229 | 343 | 345 | 205 | 211 | 108 | 108 | 402 | 412 | 336 | 346 | 421 | 449 | 299 | 301 | 158 | 164 |
| BA_MJ4405 | Mys | 122 | 128 | 72 | 86 | 193 | 221 | 119 | 141 | 170 | 172 | 217 | 217 | 345 | 345 | 209 | 211 | 126 | 128 | 412 | 438 | 318 | 334 | 421 | 447 | 279 | 301 | 146 | 156 |
| BA_MJ4401 | Mys | 136 | 136 | 100 | 100 | 207 | 221 | 143 | 147 | 174 | 174 | 229 | 229 | 345 | 345 | 201 | 201 | 126 | 142 | 404 | 410 | 348 | 348 | 435 | 447 | 303 | 307 | 154 | 160 |
| BA_MJ4400 | Mys | 130 | 132 | 72 | 94 | 223 | 223 | 129 | 135 | 174 | 178 | 219 | 219 | 345 | 345 | 207 | 211 | 126 | 128 | 404 | 416 | 318 | 342 | 433 | 449 | 289 | 295 | 154 | 156 |
| BA_MA4970 | Mys | 122 | 122 | 88 | 96 | 193 | 213 | 129 | 141 | 170 | 174 | 225 | 225 | 343 | 345 | 207 | 211 | 110 | 128 | 402 | 410 | 336 | 344 | 421 | 423 | 289 | 301 | 146 | 162 |
| BA_MA4967 | Mys | 122 | 140 | 92 | 98 | 207 | 209 | 141 | 147 | 170 | 176 | 217 | 229 | 343 | 343 | 203 | 211 | 136 | 138 | 404 | 406 | 340 | 346 | 435 | 437 | 301 | 307 | 160 | 170 |
| BA_MA4965 | Mys | 132 | 138 | 92 | 94 | 193 | 207 | 119 | 131 | 170 | 170 | 219 | 219 | 345 | 345 | 201 | 203 | 110 | 110 | 384 | 390 | 340 | 342 | 421 | 435 | 279 | 291 | 134 | 162 |
| BA_MA4477 | Mys | 134 | 138 | 70 | 70 | 193 | 219 | 129 | 135 | 168 | 176 | 217 | 217 | 343 | 347 | 187 | 203 | 128 | 138 | 406 | 426 | 318 | 318 | 421 | 445 | 289 | 295 | 152 | 156 |
| BA_MA4476 | Mys | 134 | 142 | 92 | 98 | 217 | 221 | 119 | 129 | 168 | 176 | 223 | 227 | 345 | 345 | 203 | 207 | 110 | 126 | 400 | 410 | 340 | 346 | 443 | 447 | 279 | 289 | 140 | 160 |
| BA_MA4475 | Mys | 124 | 142 | 92 | 98 | 213 | 223 | 119 | 131 | 170 | 170 | 225 | 227 | 343 | 345 | 201 | 209 | 136 | 138 | 394 | 410 | 340 | 346 | 439 | 449 | 279 | 291 | 142 | 154 |
| BA_FJ4409 | Mys | 122 | 130 | 94 | 94 | 199 | 209 | 117 | 129 | 170 | 176 | 217 | 221 | 343 | 343 | 211 | 217 | 126 | 128 | 402 | 404 | 342 | 342 | 409 | 437 | 277 | 289 | 140 | 158 |
| BA_FJ4407 | Mys | 132 | 160 | 92 | 94 | 193 | 199 | 117 | 139 | 164 | 168 | 213 | 217 | 345 | 345 | 203 | 211 | 108 | 142 | 404 | 422 | 338 | 342 | 407 | 421 | 277 | 299 | 138 | 160 |
| BA_FJ4404 | Mys | 134 | 146 | 72 | 100 | 203 | 207 | 121 | 147 | 168 | 172 | 221 | 221 | 345 | 345 | 203 | 213 | 128 | 140 | 400 | 412 | 318 | 348 | 431 | 435 | 281 | 307 | 140 | 156 |
| BA_FJ4403 | Mys | 138 | 142 | 92 | 92 | 195 | 221 | 121 | 147 | 164 | 170 | 213 | 225 | 343 | 345 | 203 | 213 | 110 | 126 | 404 | 410 | 340 | 340 | 421 | 447 | 281 | 307 | 154 | 156 |
| BA_FA4402 | Mys | 130 | 132 | 94 | 100 | 197 | 215 | 135 | 147 | 170 | 170 | 217 | 221 | 345 | 345 | 203 | 203 | 138 | 138 | 406 | 426 | 342 | 348 | 407 | 425 | 295 | 307 | 156 | 160 |
| DS_MJ4472 | Mys | 142 | 144 | 92 | 98 | 217 | 219 | 119 | 119 | 170 | 170 | 225 | 225 | 345 | 345 | 209 | 211 | 126 | 140 | 396 | 430 | 340 | 346 | 443 | 445 | 279 | 281 | 160 | 162 |
| DS_MJ4471 | Mys | 128 | 132 | 92 | 94 | 193 | 207 | 125 | 147 | 170 | 170 | 217 | 223 | 345 | 345 | 201 | 203 | 128 | 128 | 412 | 416 | 340 | 342 | 421 | 435 | 285 | 307 | 160 | 160 |
| DS_FJ4473 | Mys | 120 | 138 | 98 | 100 | 193 | 193 | 131 | 141 | 170 | 170 | 213 | 215 | 345 | 345 | 205 | 211 | 128 | 128 | 406 | 410 | 344 | 348 | 421 | 421 | 291 | 301 | 152 | 160 |
| BA_FA4957 | Bra | 126 | 134 | 110 | 110 | 205 | 213 | 129 | 131 | 170 | 170 | 223 | 223 | 341 | 357 | 197 | 215 | 102 | 102 | 372 | 372 | 356 | 356 | 431 | 439 | 289 | 291 | 142 | 156 |
| BA_FA4958 | Bra | 128 | 130 | 110 | 122 | 213 | 221 | 131 | 137 | 170 | 184 | 213 | 213 | 341 | 341 | 213 | 213 | 100 | 124 | 388 | 394 | 356 | 356 | 439 | 447 | 291 | 297 | 142 | 152 |
| BA_FA4961 | Bra | 122 | 138 | 110 | 134 | 219 | 223 | 129 | 139 | 170 | 170 | 215 | 247 | 341 | 341 | 207 | 215 | 102 | 120 | 378 | 388 | 356 | 378 | 445 | 449 | 289 | 299 | 154 | 154 |
| BA_FJ4966 | Bra | 136 | 136 | 116 | 116 | 205 | 213 | 133 | 135 | 170 | 170 | 215 | 215 | 341 | 345 | 199 | 213 | 124 | 132 | 386 | 396 | 350 | 362 | 431 | 439 | 293 | 295 | 152 | 160 |
| BA_M04962 | Bra | 124 | 132 | 114 | 146 | 207 | 209 | 129 | 133 | 170 | 170 | 213 | 221 | 341 | 341 | 205 | 215 | 104 | 116 | 372 | 398 | 346 | 390 | 433 | 435 | 289 | 293 | 156 | 162 |
| BA_MA4959 | Bra | 128 | 136 | 110 | 122 | 213 | 223 | 127 | 129 | 170 | 170 | 217 | 227 | 347 | 357 | 199 | 215 | 110 | 120 | 378 | 402 | 356 | 356 | 439 | 449 | 287 | 289 | 142 | 154 |
| BA_MA4960 | Bra | 130 | 134 | 116 | 142 | 207 | 213 | 133 | 133 | 170 | 170 | 211 | 211 | 343 | 343 | 199 | 201 | 80 | 90 | 384 | 390 | 362 | 386 | 433 | 439 | 293 | 293 | 154 | 156 |
| BA_MA4963 | Bra | 120 | 132 | 104 | 116 | 213 | 219 | 131 | 131 | 170 | 170 | 213 | 217 | 341 | 341 | 197 | 215 | 120 | 130 | 372 | 396 | 350 | 362 | 439 | 445 | 281 | 291 | 158 | 162 |
| BA_MA4964 | Bra | 136 | 138 | 110 | 128 | 215 | 221 | 131 | 137 | 170 | 170 | 217 | 217 | 341 | 357 | 203 | 205 | 100 | 104 | 378 | 396 | 356 | 362 | 441 | 447 | 291 | 297 | 154 | 156 |
| BA_MA4968 | Bra | 128 | 132 | 116 | 116 | 191 | 221 | 133 | 135 | 170 | 170 | 219 | 219 | 341 | 357 | 201 | 215 | 104 | 124 | 378 | 398 | 344 | 356 | 431 | 447 | 293 | 295 | 156 | 156 |
| BA_MJ4969 | Bra | 130 | 130 | 122 | 134 | 213 | 213 | 127 | 131 | 170 | 170 | 213 | 219 | 341 | 357 | 203 | 215 | 110 | 124 | 376 | 396 | 366 | 378 | 435 | 439 | 287 | 291 | 154 | 154 |
| SB_FA4998 | Bra | 130 | 130 | 110 | 128 | 215 | 221 | 129 | 131 | 170 | 198 | 215 | 215 | 341 | 341 | 201 | 211 | 110 | 112 | 374 | 400 | 360 | 374 | 439 | 447 | 289 | 291 | 160 | 160 |
| SB_FA5000 | Bra | 130 | 132 | 110 | 116 | 205 | 207 | 129 | 131 | 168 | 170 | 217 | 243 | 341 | 341 | 209 | 209 | 102 | 102 | 378 | 394 | 356 | 362 | 429 | 431 | 289 | 291 | 154 | 156 |
| SB_FA5001 | Bra | 138 | 138 | 104 | 122 | 209 | 215 | 129 | 135 | 172 | 184 | 211 | 217 | 341 | 341 | 199 | 211 | 106 | 122 | 364 | 396 | 350 | 356 | 433 | 439 | 289 | 295 | 152 | 154 |
| SB_MA5002 | Bra | 134 | 134 | 110 | 116 | 215 | 215 | 125 | 133 | 170 | 208 | 201 | 201 | 341 | 341 | 201 | 203 | 106 | 120 | 388 | 398 | 356 | 362 | 441 | 441 | 285 | 293 | 154 | 158 |
| SB_MA5003 | Bra | 126 | 132 | 110 | 110 | 215 | 219 | 133 | 135 | 170 | 184 | 213 | 213 | 355 | 355 | 203 | 211 | 102 | 124 | 378 | 392 | 356 | 356 | 445 | 445 | 293 | 295 | 154 | 160 |
| SB_MA5004 | Bra | 130 | 132 | 104 | 110 | 205 | 213 | 129 | 133 | 170 | 170 | 213 | 213 | 341 | 357 | 199 | 213 | 102 | 120 | 378 | 386 | 350 | 356 | 431 | 439 | 289 | 293 | 142 | 156 |
| BI_FA4980 | Bra | 130 | 134 | 116 | 116 | 213 | 217 | 131 | 133 | 170 | 170 | 217 | 217 | 341 | 347 | 201 | 211 | 112 | 132 | 398 | 398 | 356 | 356 | 439 | 443 | 291 | 293 | 154 | 156 |
| BI_MA4971 | Bra | 126 | 126 | 122 | 140 | 205 | 215 | 131 | 131 | 170 | 170 | 211 | 217 | 341 | 341 | 203 | 213 | 100 | 102 | 376 | 398 | 356 | 386 | 431 | 441 | 291 | 291 | 156 | 158 |
| BI_MA4972 | Bra | 136 | 140 | 110 | 122 | 213 | 213 | 127 | 131 | 170 | 170 | 221 | 221 | 341 | 341 | 197 | 201 | 102 | 112 | 372 | 378 | 356 | 356 | 439 | 439 | 287 | 291 | 142 | 156 |
| BI_MA4973 | Bra | 122 | 138 | 116 | 122 | 215 | 215 | 129 | 131 | 170 | 170 | 213 | 213 | 341 | 341 | 203 | 209 | 102 | 120 | 372 | 384 | 356 | 362 | 441 | 441 | 289 | 291 | 152 | 152 |
| BI_MA4978 | Bra | 134 | 134 | 110 | 116 | 215 | 225 | 127 | 129 | 170 | 170 | 217 | 219 | 341 | 341 | 205 | 215 | 124 | 134 | 372 | 394 | 356 | 356 | 441 | 449 | 287 | 289 | 154 | 154 |
| BI_MA4979 | Bra | 128 | 136 | 110 | 132 | 213 | 225 | 131 | 131 | 170 | 170 | 243 | 245 | 341 | 341 | 205 | 217 | 102 | 102 | 372 | 386 | 356 | 378 | 439 | 449 | 291 | 291 | 154 | 154 |
| BI_MJ4974 | Bra | 126 | 132 | 110 | 116 | 207 | 217 | 133 | 135 | 170 | 170 | 215 | 247 | 341 | 341 | 203 | 205 | 102 | 108 | 392 | 392 | 356 | 362 | 433 | 447 | 293 | 295 | 154 | 154 |
| BI_MJ4981 | Bra | 130 | 130 | 110 | 116 | 215 | 217 | 125 | 125 | 170 | 170 | 215 | 221 | 341 | 341 | 201 | 209 | 102 | 120 | 378 | 396 | 344 | 356 | 441 | 443 | 285 | 285 | 156 | 158 |
| BN_MJ4458 | Bra | 130 | 130 | 110 | 110 | 213 | 223 | 127 | 135 | 170 | 178 | 217 | 217 | 341 | 341 | 201 | 213 | 100 | 102 | 376 | 392 | 356 | 356 | 439 | 447 | 287 | 295 | 154 | 156 |
| BN_MJ4459 | Bra | 130 | 134 | 122 | 122 | 205 | 223 | 127 | 127 | 170 | 170 | 223 | 223 | 341 | 341 | 201 | 205 | 100 | 128 | 398 | 398 | 356 | 378 | 431 | 449 | 281 | 287 | 156 | 158 |
| BN_MA4461 | Bra | 120 | 134 | 128 | 134 | 205 | 223 | 117 | 131 | 170 | 172 | 217 | 223 | 341 | 341 | 201 | 215 | 102 | 130 | 366 | 400 | 374 | 378 | 431 | 449 | 277 | 291 | 154 | 158 |
| BN_MA4462 | Bra | 130 | 130 | 110 | 110 | 213 | 221 | 127 | 135 | 170 | 178 | 217 | 217 | 341 | 341 | 201 | 215 | 100 | 102 | 376 | 394 | 352 | 352 | 441 | 447 | 285 | 293 | 154 | 156 |
| BN_MA4909 | Bra | 128 | 130 | 110 | 128 | 215 | 225 | 129 | 135 | 170 | 172 | 213 | 213 | 341 | 341 | 203 | 209 | 102 | 102 | 388 | 396 | 342 | 362 | 441 | 449 | 289 | 295 | 152 | 154 |
| BS_M04645 | Bra | 132 | 136 | 110 | 122 | 205 | 215 | 125 | 125 | 170 | 172 | 233 | 243 | 341 | 341 | 201 | 203 | 102 | 124 | 378 | 392 | 356 | 368 | 431 | 441 | 281 | 285 | 156 | 158 |
| BS_M04646 | Bra | 134 | 136 | 110 | 110 | 217 | 221 | 127 | 129 | 184 | 204 | 217 | 223 | 341 | 341 | 213 | 213 | 124 | 124 | 376 | 398 | 356 | 356 | 441 | 445 | 287 | 289 | 152 | 156 |
| BS_MA2461 | Bra | 124 | 126 | 110 | 128 | 213 | 219 | 133 | 133 | 184 | 192 | 217 | 229 | 345 | 345 | 205 | 211 | 108 | 128 | 378 | 394 | 356 | 374 | 439 | 445 | 293 | 295 | 138 | 154 |
| BS_MA2462 | Bra | 126 | 134 | 110 | 140 | 213 | 215 | 123 | 125 | 172 | 172 | 217 | 217 | 343 | 343 | 205 | 211 | 100 | 108 | 372 | 372 | 356 | 386 | 439 | 441 | 283 | 285 | 152 | 162 |
| BS_MA2464 | Bra | 130 | 138 | 110 | 116 | 213 | 215 | 129 | 129 | 170 | 170 | 215 | 215 | 345 | 345 | 211 | 211 | 128 | 128 | 378 | 392 | 356 | 362 | 439 | 441 | 289 | 289 | 154 | 156 |
| BS_MA2467 | Bra | 132 | 136 | 110 | 130 | 213 | 219 | 129 | 135 | 172 | 172 | 219 | 219 | 341 | 341 | 197 | 213 | 110 | 122 | 392 | 398 | 356 | 364 | 439 | 445 | 289 | 295 | 152 | 158 |
| BS_MA4910 | Bra | 124 | 130 | 110 | 122 | 205 | 207 | 127 | 137 | 170 | 170 | 243 | 243 | 341 | 341 | 203 | 207 | 102 | 134 | 378 | 388 | 356 | 356 | 431 | 433 | 287 | 297 | 142 | 154 |
| BS_MA4914 | Bra | 130 | 132 | 110 | 110 | 207 | 207 | 131 | 139 | 172 | 200 | 213 | 217 | 341 | 341 | 201 | 205 | 102 | 134 | 398 | 398 | 344 | 354 | 433 | 433 | 291 | 299 | 154 | 154 |
| BS_MJ4908 | Bra | 122 | 128 | 164 | 164 | 213 | 215 | 127 | 127 | 170 | 184 | 217 | 221 | 341 | 341 | 203 | 215 | 104 | 120 | 388 | 390 | 408 | 408 | 439 | 439 | 281 | 287 | 154 | 162 |
| BS_MJ4911 | Bra | 130 | 134 | 128 | 128 | 205 | 209 | 131 | 137 | 170 | 170 | 217 | 243 | 341 | 357 | 201 | 203 | 102 | 122 | 372 | 378 | 362 | 362 | 431 | 435 | 291 | 297 | 154 | 154 |
| BS_MJ4913 | Bra | 132 | 138 | 110 | 110 | 215 | 221 | 133 | 137 | 170 | 184 | 219 | 219 | 341 | 341 | 199 | 205 | 100 | 102 | 378 | 390 | 356 | 356 | 441 | 447 | 293 | 297 | 138 | 158 |
| BW_FA4451 | Bra | 124 | 132 | 110 | 122 | 207 | 207 | 127 | 129 | 170 | 170 | 215 | 215 | 341 | 341 | 197 | 213 | 120 | 124 | 378 | 392 | 356 | 356 | 433 | 433 | 287 | 289 | 152 | 152 |
| BW_FA4878 | Bra | 130 | 138 | 110 | 134 | 219 | 221 | 117 | 133 | 202 | 214 | 213 | 217 | 341 | 341 | 201 | 213 | 102 | 104 | 376 | 398 | 356 | 380 | 445 | 447 | 277 | 293 | 154 | 156 |
| BW_FA4888 | Bra | 120 | 130 | 116 | 128 | 207 | 213 | 125 | 133 | 170 | 172 | 217 | 241 | 341 | 341 | 211 | 213 | 102 | 102 | 386 | 392 | 362 | 374 | 433 | 439 | 285 | 293 | 152 | 154 |
| BW_FJ4890 | Bra | 126 | 130 | 110 | 130 | 213 | 213 | 127 | 137 | 170 | 172 | 213 | 247 | 341 | 341 | 199 | 213 | 102 | 130 | 376 | 376 | 356 | 374 | 439 | 439 | 287 | 297 | 154 | 156 |
| BW_M04649 | Bra | 128 | 134 | 116 | 134 | 217 | 223 | 133 | 135 | 170 | 170 | 217 | 219 | 341 | 341 | 203 | 213 | 102 | 120 | 372 | 398 | 362 | 378 | 441 | 447 | 293 | 295 | 154 | 158 |
| BW_M04650 | Bra | 130 | 138 | 122 | 130 | 221 | 221 | 127 | 129 | 170 | 184 | 203 | 203 | 341 | 341 | 213 | 215 | 102 | 132 | 378 | 396 | 368 | 378 | 447 | 447 | 287 | 289 | 154 | 154 |
| BW_M04651 | Bra | 130 | 130 | 110 | 114 | 207 | 227 | 131 | 137 | 170 | 170 | 205 | 205 | 341 | 341 | 197 | 211 | 100 | 100 | 386 | 392 | 348 | 358 | 431 | 453 | 291 | 297 | 156 | 158 |
| BW_MA4449 | Bra | 134 | 140 | 110 | 134 | 213 | 221 | 129 | 129 | 170 | 172 | 215 | 217 | 341 | 341 | 211 | 213 | 102 | 104 | 372 | 372 | 356 | 378 | 439 | 447 | 289 | 289 | 154 | 156 |
| BW_MA4867 | Bra | 112 | 132 | 116 | 130 | 203 | 213 | 131 | 135 | 170 | 170 | 213 | 213 | 341 | 341 | 197 | 213 | 102 | 106 | 386 | 392 | 362 | 376 | 429 | 439 | 291 | 295 | 152 | 162 |
| BW_MA4876 | Bra | 130 | 130 | 116 | 122 | 205 | 219 | 129 | 133 | 170 | 192 | 203 | 213 | 341 | 341 | 201 | 201 | 102 | 106 | 376 | 390 | 356 | 362 | 431 | 445 | 289 | 293 | 154 | 156 |
| BW_MA4877 | Bra | 132 | 136 | 110 | 116 | 207 | 213 | 131 | 135 | 170 | 170 | 221 | 223 | 341 | 341 | 203 | 203 | 102 | 106 | 374 | 376 | 356 | 362 | 435 | 439 | 291 | 295 | 152 | 154 |
| BW_MA4879 | Bra | 130 | 132 | 110 | 112 | 223 | 223 | 135 | 137 | 172 | 192 | 205 | 219 | 341 | 341 | 203 | 211 | 102 | 108 | 372 | 398 | 356 | 356 | 435 | 449 | 295 | 297 | 152 | 154 |
| BW_MA4880 | Bra | 126 | 130 | 110 | 122 | 213 | 213 | 137 | 137 | 184 | 184 | 215 | 215 | 341 | 341 | 201 | 203 | 102 | 134 | 378 | 396 | 356 | 356 | 439 | 439 | 297 | 297 | 154 | 160 |
| BW_MA4881 | Bra | 132 | 138 | 114 | 116 | 213 | 213 | 127 | 133 | 170 | 194 | 217 | 217 | 341 | 357 | 201 | 211 | 102 | 106 | 386 | 392 | 346 | 362 | 435 | 439 | 287 | 293 | 152 | 158 |
| BW_MA4884 | Bra | 130 | 134 | 134 | 164 | 201 | 217 | 127 | 131 | 170 | 170 | 213 | 217 | 341 | 341 | 199 | 201 | 104 | 122 | 372 | 384 | 378 | 410 | 427 | 443 | 287 | 291 | 142 | 154 |
| BW_MA4885 | Bra | 118 | 122 | 128 | 128 | 203 | 213 | 131 | 131 | 170 | 170 | 213 | 215 | 341 | 357 | 203 | 205 | 106 | 120 | 378 | 378 | 362 | 362 | 429 | 439 | 291 | 291 | 156 | 158 |
| BW_MA4886 | Bra | 130 | 136 | 110 | 116 | 213 | 219 | 123 | 133 | 170 | 186 | 213 | 213 | 341 | 341 | 197 | 205 | 100 | 102 | 376 | 386 | 356 | 362 | 439 | 445 | 283 | 293 | 156 | 158 |
| BW_MA4887 | Bra | 128 | 134 | 104 | 110 | 207 | 207 | 133 | 133 | 170 | 206 | 213 | 243 | 341 | 341 | 203 | 213 | 100 | 130 | 390 | 396 | 350 | 356 | 433 | 435 | 281 | 293 | 158 | 162 |
| BW_MA4891 | Bra | 124 | 130 | 110 | 128 | 219 | 219 | 127 | 133 | 170 | 184 | 213 | 223 | 341 | 341 | 201 | 209 | 104 | 106 | 378 | 378 | 344 | 362 | 445 | 445 | 287 | 293 | 154 | 156 |
| BW_MA4946 | Bra | 132 | 132 | 110 | 122 | 215 | 215 | 131 | 133 | 170 | 170 | 219 | 223 | 341 | 341 | 201 | 201 | 120 | 120 | 376 | 384 | 356 | 356 | 433 | 441 | 291 | 293 | 154 | 156 |
| BW_MJ4889 | Bra | 128 | 134 | 110 | 110 | 203 | 215 | 129 | 129 | 170 | 170 | 217 | 225 | 341 | 341 | 197 | 203 | 102 | 104 | 400 | 402 | 356 | 356 | 429 | 439 | 281 | 289 | 152 | 154 |
| GO_MJ4943 | Bra | 134 | 138 | 110 | 116 | 219 | 223 | 129 | 135 | 170 | 172 | 213 | 213 | 343 | 343 | 203 | 205 | 102 | 122 | 384 | 394 | 344 | 362 | 445 | 449 | 289 | 295 | 154 | 154 |
| GO_MJ4944 | Bra | 128 | 128 | 116 | 116 | 213 | 217 | 133 | 139 | 170 | 170 | 215 | 215 | 341 | 341 | 207 | 213 | 102 | 110 | 384 | 400 | 362 | 362 | 439 | 443 | 293 | 299 | 154 | 156 |
| GO_MJ4945 | Bra | 132 | 134 | 120 | 132 | 213 | 213 | 137 | 143 | 170 | 190 | 217 | 243 | 341 | 341 | 201 | 201 | 100 | 104 | 384 | 388 | 366 | 378 | 435 | 439 | 295 | 303 | 154 | 166 |
| GO_FA4949 | Bra | 122 | 134 | 122 | 140 | 207 | 213 | 131 | 131 | 170 | 172 | 217 | 223 | 341 | 341 | 203 | 211 | 102 | 122 | 376 | 400 | 356 | 386 | 433 | 439 | 291 | 291 | 142 | 158 |
| GO_FJ4948 | Bra | 134 | 136 | 110 | 122 | 201 | 217 | 127 | 137 | 170 | 182 | 213 | 213 | 341 | 341 | 205 | 215 | 104 | 120 | 392 | 400 | 356 | 368 | 427 | 443 | 287 | 297 | 142 | 154 |
| GO_MA4935 | Bra | 130 | 134 | 122 | 132 | 215 | 219 | 133 | 139 | 170 | 170 | 213 | 213 | 341 | 341 | 197 | 201 | 102 | 102 | 384 | 400 | 368 | 378 | 441 | 445 | 293 | 299 | 154 | 162 |
| GO_MA4940 | Bra | 130 | 130 | 110 | 122 | 215 | 221 | 125 | 125 | 170 | 172 | 215 | 215 | 341 | 347 | 209 | 213 | 102 | 102 | 384 | 396 | 356 | 368 | 441 | 447 | 285 | 285 | 156 | 158 |
| GO_MA4942 | Bra | 130 | 132 | 116 | 140 | 207 | 217 | 127 | 131 | 170 | 170 | 223 | 223 | 341 | 341 | 197 | 205 | 100 | 130 | 378 | 388 | 362 | 386 | 433 | 443 | 287 | 291 | 158 | 160 |
| GO_MA4947 | Bra | 130 | 134 | 110 | 110 | 205 | 207 | 133 | 133 | 170 | 172 | 213 | 217 | 341 | 341 | 199 | 215 | 110 | 122 | 376 | 400 | 356 | 356 | 431 | 433 | 293 | 293 | 152 | 154 |
| GO_MA4952 | Bra | 130 | 130 | 110 | 134 | 213 | 219 | 129 | 129 | 170 | 170 | 217 | 217 | 341 | 341 | 201 | 213 | 102 | 102 | 378 | 394 | 356 | 378 | 439 | 445 | 281 | 289 | 156 | 156 |
| GO_MA4956 | Bra | 126 | 126 | 110 | 148 | 213 | 221 | 131 | 131 | 172 | 174 | 213 | 217 | 341 | 341 | 197 | 209 | 102 | 126 | 376 | 378 | 356 | 394 | 439 | 447 | 291 | 291 | 152 | 166 |
| GO_MJ4446 | Bra | 132 | 132 | 110 | 110 | 213 | 213 | 135 | 135 | 170 | 202 | 217 | 247 | 341 | 341 | 201 | 211 | 100 | 102 | 372 | 400 | 356 | 372 | 439 | 439 | 281 | 295 | 158 | 160 |
| DS_FA4918 | Bra | 128 | 138 | 110 | 110 | 215 | 219 | 127 | 129 | 172 | 198 | 217 | 245 | 341 | 341 | 207 | 213 | 122 | 122 | 372 | 398 | 356 | 378 | 441 | 445 | 287 | 289 | 156 | 170 |
| DS_FJ4919 | Bra | 122 | 128 | 110 | 110 | 205 | 221 | 123 | 133 | 170 | 170 | 217 | 217 | 341 | 341 | 205 | 213 | 102 | 102 | 372 | 376 | 356 | 356 | 431 | 445 | 283 | 293 | 154 | 160 |
| DS_FJ4921 | Bra | 128 | 132 | 122 | 140 | 207 | 215 | 131 | 133 | 170 | 172 | 217 | 217 | 341 | 341 | 197 | 197 | 102 | 106 | 386 | 398 | 368 | 386 | 433 | 441 | 291 | 293 | 156 | 156 |
| DS_FJ4923 | Bra | 136 | 138 | 110 | 110 | 217 | 219 | 129 | 135 | 172 | 172 | 221 | 221 | 343 | 355 | 199 | 207 | 100 | 110 | 372 | 392 | 356 | 356 | 443 | 445 | 289 | 295 | 156 | 158 |
| DS_FJ4933 | Bra | 128 | 132 | 122 | 140 | 207 | 215 | 131 | 133 | 170 | 172 | 217 | 217 | 345 | 345 | 199 | 199 | 104 | 108 | 386 | 398 | 368 | 386 | 433 | 441 | 291 | 293 | 156 | 156 |
| DS_MA4917 | Bra | 124 | 130 | 104 | 122 | 205 | 205 | 133 | 135 | 170 | 198 | 221 | 221 | 341 | 341 | 209 | 217 | 106 | 120 | 376 | 378 | 350 | 356 | 431 | 431 | 293 | 295 | 154 | 158 |
| DS_MA4920 | Bra | 130 | 132 | 110 | 122 | 205 | 217 | 127 | 135 | 170 | 170 | 215 | 239 | 341 | 341 | 203 | 203 | 102 | 122 | 372 | 384 | 356 | 356 | 431 | 443 | 287 | 295 | 154 | 160 |
| DS_MA4924 | Bra | 132 | 136 | 122 | 134 | 207 | 213 | 129 | 131 | 170 | 198 | 225 | 225 | 343 | 343 | 201 | 201 | 100 | 122 | 378 | 398 | 354 | 378 | 431 | 439 | 289 | 291 | 160 | 160 |
| DS_MA4925 | Bra | 122 | 128 | 110 | 110 | 213 | 215 | 131 | 135 | 170 | 170 | 215 | 215 | 341 | 341 | 201 | 207 | 120 | 122 | 384 | 392 | 342 | 356 | 439 | 441 | 291 | 295 | 154 | 158 |
| DS_MA4926 | Bra | 130 | 132 | 128 | 128 | 215 | 223 | 133 | 135 | 170 | 170 | 223 | 223 | 343 | 343 | 203 | 205 | 104 | 122 | 392 | 396 | 362 | 362 | 441 | 449 | 293 | 295 | 152 | 154 |
| DS_MA4927 | Bra | 120 | 130 | 110 | 110 | 209 | 225 | 127 | 131 | 170 | 170 | 213 | 213 | 343 | 359 | 199 | 205 | 102 | 108 | 372 | 384 | 356 | 356 | 435 | 451 | 287 | 291 | 138 | 158 |
| DS_MA4928 | Bra | 132 | 136 | 98 | 110 | 207 | 215 | 127 | 131 | 170 | 170 | 217 | 245 | 343 | 343 | 199 | 201 | 122 | 130 | 382 | 398 | 344 | 356 | 433 | 441 | 287 | 291 | 154 | 158 |
| DS_MA4929 | Bra | 128 | 134 | 110 | 128 | 213 | 229 | 131 | 135 | 170 | 172 | 217 | 217 | 343 | 359 | 203 | 205 | 104 | 112 | 388 | 392 | 356 | 374 | 439 | 455 | 291 | 295 | 154 | 156 |
| DS_MJ4915 | Bra | 130 | 138 | 110 | 122 | 207 | 213 | 129 | 135 | 170 | 184 | 217 | 217 | 341 | 341 | 199 | 211 | 102 | 132 | 372 | 394 | 344 | 356 | 431 | 439 | 289 | 295 | 158 | 162 |
| DS_MJ4916 | Bra | 130 | 136 | 110 | 128 | 221 | 225 | 131 | 135 | 170 | 194 | 213 | 221 | 341 | 357 | 203 | 209 | 102 | 102 | 388 | 400 | 356 | 362 | 447 | 449 | 291 | 295 | 162 | 168 |
| DS_MJ4922 | Bra | 120 | 130 | 110 | 110 | 215 | 219 | 133 | 135 | 184 | 202 | 201 | 201 | 343 | 343 | 199 | 207 | 124 | 124 | 378 | 390 | 356 | 356 | 441 | 445 | 293 | 295 | 142 | 154 |
| DS_MJ4932 | Bra | 122 | 140 | 122 | 140 | 201 | 221 | 133 | 135 | 170 | 170 | 217 | 223 | 343 | 343 | 203 | 211 | 110 | 122 | 378 | 394 | 356 | 386 | 427 | 445 | 293 | 295 | 152 | 156 |
| PC_FA4905 | Bra | 130 | 140 | 110 | 110 | 213 | 219 | 127 | 133 | 170 | 186 | 221 | 221 | 341 | 341 | 197 | 211 | 100 | 100 | 384 | 398 | 356 | 356 | 439 | 445 | 287 | 293 | 154 | 158 |
| PC_FA4907 | Bra | 128 | 130 | 126 | 138 | 213 | 219 | 135 | 135 | 170 | 170 | 201 | 201 | 341 | 341 | 203 | 211 | 106 | 124 | 376 | 400 | 372 | 382 | 439 | 445 | 295 | 295 | 152 | 162 |
| PC_MA4906 | Bra | 128 | 136 | 116 | 128 | 205 | 219 | 127 | 133 | 170 | 194 | 215 | 217 | 341 | 341 | 199 | 203 | 120 | 120 | 378 | 392 | 362 | 362 | 431 | 445 | 287 | 293 | 138 | 154 |
| PC_MJ4994 | Bra | 124 | 132 | 116 | 128 | 215 | 217 | 127 | 135 | 170 | 192 | 217 | 223 | 341 | 341 | 213 | 215 | 102 | 114 | 382 | 388 | 362 | 362 | 441 | 443 | 287 | 295 | 154 | 158 |
| PC_MJ4995 | Bra | 130 | 140 | 110 | 120 | 203 | 211 | 131 | 135 | 170 | 170 | 213 | 219 | 341 | 341 | 205 | 205 | 112 | 134 | 378 | 388 | 356 | 366 | 427 | 435 | 291 | 295 | 154 | 154 |
| PC_MJ4996 | Bra | 130 | 138 | 110 | 110 | 209 | 209 | 125 | 135 | 170 | 170 | 213 | 213 | 341 | 357 | 201 | 203 | 100 | 106 | 378 | 378 | 356 | 356 | 433 | 433 | 285 | 295 | 158 | 162 |
| PC_MJ4997 | Bra | 130 | 134 | 110 | 132 | 215 | 223 | 133 | 141 | 170 | 170 | 213 | 219 | 341 | 341 | 197 | 207 | 110 | 120 | 394 | 398 | 356 | 378 | 439 | 449 | 293 | 301 | 142 | 154 |
| TR_M04662 | Bra | 126 | 134 | 110 | 110 | 215 | 215 | 135 | 137 | 170 | 170 | 213 | 239 | 341 | 341 | 207 | 215 | 102 | 120 | 386 | 392 | 356 | 356 | 441 | 441 | 295 | 297 | 154 | 160 |
| TR_MA4868 | Bra | 132 | 132 | 110 | 110 | 205 | 213 | 129 | 133 | 184 | 192 | 221 | 233 | 341 | 341 | 199 | 207 | 120 | 120 | 366 | 378 | 356 | 356 | 431 | 439 | 289 | 293 | 152 | 164 |
| TR_MA4869 | Bra | 130 | 138 | 110 | 116 | 213 | 213 | 127 | 129 | 170 | 184 | 217 | 217 | 341 | 341 | 201 | 215 | 102 | 102 | 372 | 372 | 344 | 362 | 435 | 439 | 287 | 289 | 152 | 154 |
| TR_MA4870 | Bra | 134 | 138 | 114 | 116 | 213 | 219 | 131 | 133 | 170 | 170 | 213 | 243 | 341 | 341 | 197 | 197 | 102 | 124 | 378 | 396 | 360 | 362 | 439 | 445 | 291 | 291 | 160 | 160 |
| TR_MA4871 | Bra | 132 | 132 | 110 | 110 | 207 | 221 | 135 | 141 | 170 | 184 | 221 | 223 | 341 | 341 | 199 | 205 | 104 | 104 | 388 | 392 | 356 | 356 | 433 | 447 | 295 | 301 | 144 | 160 |
| TR_MA4872 | Bra | 134 | 138 | 110 | 110 | 207 | 221 | 133 | 133 | 170 | 184 | 223 | 243 | 341 | 341 | 203 | 215 | 106 | 108 | 376 | 378 | 356 | 378 | 433 | 447 | 293 | 293 | 142 | 154 |
| TR_MA4892 | Bra | 132 | 134 | 110 | 126 | 205 | 225 | 133 | 135 | 170 | 184 | 215 | 221 | 341 | 341 | 205 | 209 | 100 | 106 | 372 | 394 | 356 | 372 | 431 | 449 | 293 | 295 | 154 | 158 |
| TR_MA4893 | Bra | 130 | 132 | 110 | 110 | 213 | 219 | 133 | 133 | 170 | 212 | 223 | 223 | 341 | 357 | 199 | 203 | 102 | 120 | 376 | 382 | 356 | 356 | 439 | 445 | 293 | 293 | 156 | 160 |
| TR_MA4894 | Bra | 134 | 136 | 110 | 110 | 221 | 225 | 117 | 129 | 172 | 212 | 215 | 217 | 341 | 357 | 197 | 203 | 102 | 126 | 398 | 402 | 356 | 356 | 445 | 451 | 277 | 289 | 154 | 158 |
| TR_MA4895 | Bra | 126 | 140 | 112 | 128 | 219 | 219 | 131 | 135 | 170 | 172 | 215 | 215 | 341 | 341 | 207 | 215 | 102 | 104 | 378 | 384 | 358 | 362 | 445 | 445 | 291 | 295 | 158 | 162 |
| TR_MA4896 | Bra | 124 | 128 | 110 | 134 | 215 | 219 | 131 | 131 | 170 | 172 | 211 | 215 | 341 | 341 | 197 | 207 | 102 | 106 | 388 | 392 | 356 | 378 | 441 | 445 | 281 | 291 | 154 | 156 |
| TR_MA4898 | Bra | 132 | 136 | 104 | 122 | 217 | 217 | 121 | 135 | 172 | 198 | 217 | 221 | 341 | 341 | 209 | 211 | 102 | 102 | 378 | 388 | 352 | 368 | 435 | 443 | 281 | 295 | 138 | 162 |
| TR_MA4900 | Bra | 136 | 136 | 122 | 128 | 205 | 211 | 127 | 127 | 172 | 192 | 243 | 249 | 341 | 341 | 199 | 203 | 108 | 112 | 376 | 388 | 362 | 368 | 431 | 437 | 281 | 287 | 152 | 156 |
| TR_MA4904 | Bra | 122 | 138 | 110 | 110 | 219 | 221 | 127 | 131 | 170 | 170 | 223 | 243 | 341 | 341 | 199 | 205 | 108 | 122 | 378 | 392 | 356 | 412 | 445 | 447 | 287 | 291 | 152 | 154 |
| TR_MJ4899 | Bra | 130 | 134 | 110 | 114 | 217 | 221 | 133 | 135 | 170 | 172 | 213 | 213 | 341 | 357 | 201 | 201 | 102 | 120 | 376 | 378 | 356 | 360 | 443 | 447 | 293 | 295 | 158 | 158 |
| TR_MJ4903 | Bra | 130 | 136 | 122 | 122 | 207 | 217 | 129 | 131 | 170 | 170 | 215 | 215 | 341 | 341 | 205 | 213 | 102 | 122 | 378 | 386 | 356 | 368 | 433 | 443 | 289 | 291 | 154 | 156 |
| TR_F04665 | Bra | 124 | 138 | 110 | 122 | 209 | 213 | 127 | 137 | 170 | 170 | 215 | 219 | 341 | 341 | 209 | 211 | 122 | 122 | 372 | 396 | 356 | 356 | 435 | 439 | 287 | 297 | 142 | 156 |
| TR_FA4873 | Bra | 130 | 130 | 116 | 142 | 213 | 213 | 125 | 131 | 184 | 212 | 215 | 217 | 341 | 341 | 205 | 213 | 120 | 130 | 376 | 382 | 362 | 386 | 439 | 439 | 285 | 291 | 154 | 160 |
| WW_FA4989 | Bra | 130 | 136 | 110 | 122 | 215 | 221 | 127 | 131 | 170 | 212 | 213 | 245 | 341 | 341 | 201 | 213 | 102 | 102 | 374 | 376 | 356 | 356 | 439 | 445 | 287 | 291 | 152 | 166 |
| WW_FJ4986 | Bra | 130 | 138 | 110 | 110 | - | - | - | - | 170 | 200 | 217 | 223 | 341 | 341 | 203 | 209 | 102 | 112 | 378 | 398 | 356 | 356 | 439 | 439 | 287 | 289 | 152 | 162 |
| WW_MA4983 | Bra | 130 | 144 | 122 | 122 | - | - | 131 | 131 | 170 | 190 | 217 | 221 | 341 | 341 | 203 | 203 | 102 | 114 | 374 | 376 | 356 | 368 | 441 | 441 | 281 | 291 | 154 | 158 |
| WW_MA4984 | Bra | 134 | 136 | 110 | 122 | 205 | 223 | 131 | 131 | 170 | 194 | 213 | 223 | 341 | 341 | 201 | 213 | 120 | 124 | 378 | 384 | 356 | 368 | 429 | 447 | 281 | 291 | 152 | 158 |
| WW_MA4988 | Bra | 128 | 138 | 110 | 126 | 211 | 221 | 127 | 133 | 170 | 194 | 213 | 243 | 341 | 341 | 203 | 203 | 102 | 122 | 384 | 384 | 356 | 372 | 435 | 447 | 287 | 293 | 138 | 158 |
| WW_MA4990 | Bra | 128 | 128 | 110 | 110 | 203 | 221 | 131 | 137 | 170 | 170 | 217 | 217 | 341 | 341 | 203 | 215 | 106 | 120 | 394 | 394 | 356 | 356 | 427 | 447 | 291 | 297 | 152 | 158 |
| WW_MA4991 | Bra | 132 | 134 | 122 | 164 | 219 | 221 | 129 | 129 | 170 | 170 | 217 | 217 | 341 | 341 | 203 | 209 | 112 | 122 | 376 | 392 | 356 | 410 | 445 | 447 | 279 | 289 | 154 | 156 |
| WW_MA4992 | Bra | 130 | 134 | 128 | 164 | 215 | 219 | 129 | 133 | 170 | 170 | 219 | 219 | 341 | 341 | 199 | 201 | 120 | 134 | 384 | 394 | 362 | 408 | 441 | 445 | 289 | 293 | 154 | 156 |
| WW_MJ4982 | Bra | 130 | 136 | 110 | 132 | 213 | 219 | 127 | 131 | 170 | 172 | 223 | 223 | 341 | 341 | 199 | 203 | 106 | 110 | 382 | 396 | 356 | 378 | 439 | 445 | 287 | 291 | 154 | 156 |
| WW_MJ4985 | Bra | 124 | 136 | 110 | 128 | - | - | - | - | 170 | 170 | 223 | 223 | 341 | 341 | 199 | 215 | 100 | 104 | 374 | 394 | 356 | 374 | 431 | 451 | 281 | 281 | 144 | 160 |
| WW_MJ4987 | Bra | 134 | 134 | 116 | 132 | - | - | - | - | 170 | 198 | 223 | 225 | 341 | 341 | 213 | 215 | 102 | 112 | 372 | 398 | 350 | 378 | 439 | 443 | 285 | 287 | 156 | 158 |
| WW_MJ4993 | Bra | 128 | 134 | 110 | 116 | 205 | 221 | 127 | 131 | 184 | 184 | 213 | 247 | 341 | 341 | 197 | 211 | 102 | 124 | 374 | 378 | 356 | 362 | 431 | 447 | 287 | 291 | 154 | 156 |
| PS_F02865 | Bra | 132 | 132 | 116 | 116 | 213 | 221 | 129 | 131 | 172 | 172 | 215 | 215 | 341 | 341 | 205 | 215 | 102 | 106 | 374 | 378 | 362 | 376 | 439 | 447 | 289 | 291 | 152 | 154 |
| PS_M03380 | Bra | 132 | 134 | 122 | 122 | 217 | 225 | 125 | 133 | 170 | 170 | 213 | 213 | 341 | 357 | 197 | 199 | 102 | 124 | 392 | 394 | 356 | 356 | 445 | 451 | 285 | 293 | 158 | 160 |
| WK_MA4455 | Bra | 130 | 130 | 122 | 122 | 205 | 215 | 131 | 133 | 170 | 172 | 243 | 243 | 341 | 355 | 205 | 217 | 104 | 106 | 414 | 422 | 314 | 322 | 431 | 435 | 285 | 301 | 152 | 164 |
| WK_FA4457 | Bra | 124 | 138 | 110 | 110 | 209 | 213 | 127 | 127 | 170 | 170 | 203 | 221 | 341 | 341 | 201 | 213 | 102 | 106 | 378 | 396 | 356 | 356 | 435 | 439 | 281 | 287 | 152 | 154 |
| WK_MJ5219 | Bra | 132 | 136 | 122 | 122 | 205 | 217 | 127 | 129 | 170 | 170 | 213 | 221 | 341 | 357 | 199 | 203 | 106 | 110 | 372 | 378 | 356 | 356 | 431 | 443 | 287 | 289 | 152 | 162 |
| WK_MA5222 | Bra | 128 | 138 | 110 | 122 | 213 | 213 | 127 | 135 | 170 | 170 | 209 | 215 | 341 | 341 | 197 | 197 | 108 | 122 | 372 | 396 | 356 | 356 | 439 | 439 | 287 | 295 | 154 | 154 |
| BN_M01389 | Alc | - | - | - | - | 195 | 213 | - | - | 168 | 168 | - | - | - | - | - | - | - | - | 410 | 420 | 322 | 322 | 421 | 439 | 277 | 297 | 152 | 166 |
| BN_FA5011 | Alc | 142 | 150 | 84 | 84 | - | - | 135 | 139 | 186 | 198 | 249 | 249 | 359 | 369 | 201 | 209 | 116 | 118 | 410 | 412 | 322 | 332 | 433 | 435 | 293 | 293 | 166 | 168 |
| BN_M01440 | Alc | 124 | 124 | - | - | 213 | 215 | 117 | 117 | 190 | 194 | 217 | 217 | 345 | 345 | 205 | 207 | 110 | 126 | 410 | 410 | 314 | 314 | 439 | 441 | 279 | 279 | 168 | 168 |
| BN_MA5010 | Alc | 148 | 150 | 88 | 88 | 213 | 217 | 139 | 145 | 190 | 194 | 249 | 249 | 361 | 363 | 209 | 217 | 118 | 118 | 410 | 410 | 314 | 334 | 439 | 443 | 297 | 303 | 152 | 166 |
| BN_MJ4460 | Alc | 136 | 138 | - | - | 207 | 213 | 135 | 141 | 170 | 188 | 249 | 249 | 361 | 375 | 199 | 209 | 100 | 118 | 406 | 410 | 314 | 322 | 433 | 439 | 293 | 299 | 152 | 166 |
| BN_MJ4463 | Alc | 142 | 156 | - | - | 207 | 207 | 117 | 137 | 176 | 196 | 249 | 251 | 361 | 361 | 213 | 219 | 116 | 116 | 410 | 410 | 322 | 322 | 433 | 433 | 277 | 295 | 166 | 166 |
| BI_M03550 | Alc | 150 | 150 | 92 | 92 | 205 | 207 | 133 | 147 | - | - | 249 | 249 | 359 | 383 | 199 | 201 | 116 | 118 | 410 | 410 | 314 | 340 | 431 | 433 | 291 | 305 | 158 | 166 |
| BI_MA3551 | Alc | 144 | 158 | 76 | 76 | 209 | 213 | 141 | 147 | 190 | 190 | 237 | 249 | 359 | 359 | 199 | 215 | 118 | 124 | 410 | 410 | 314 | 314 | 435 | 439 | 299 | 305 | 168 | 170 |
| BI_MA4467 | Alc | 146 | 146 | 0 | 0 | 207 | 217 | 143 | 145 | 192 | 208 | 249 | 249 | 361 | 367 | 201 | 217 | 116 | 116 | 402 | 410 | 322 | 322 | 433 | 443 | 301 | 303 | 152 | 168 |
| BI_MJ4975 | Alc | 128 | 128 | 92 | 94 | 207 | 213 | 131 | 141 | 170 | 170 | 249 | 249 | 359 | 367 | 201 | 201 | 120 | 128 | 410 | 414 | 338 | 340 | 433 | 439 | 289 | 299 | 166 | 166 |
| BI_FJ4466 | Alc | 144 | 154 | 70 | 70 | 207 | 209 | 139 | 141 | 186 | 186 | 249 | 249 | 373 | 383 | 209 | 213 | 112 | 118 | 412 | 416 | 314 | 322 | 433 | 435 | 297 | 297 | 166 | 170 |
| BI_FJ4976 | Alc | 128 | 132 | 116 | 116 | 207 | 209 | 137 | 141 | 170 | 170 | 249 | 249 | 373 | 379 | 211 | 217 | 116 | 120 | 410 | 414 | 322 | 338 | 433 | 435 | 295 | 299 | 160 | 168 |
| BI_FJ4977 | Alc | 128 | 134 | 86 | 116 | 213 | 217 | 133 | 133 | 190 | 194 | 249 | 249 | 359 | 369 | 211 | 215 | 116 | 118 | 410 | 410 | 322 | 332 | 439 | 443 | 291 | 291 | 164 | 166 |
| BW_001511 | Alc | 140 | 148 | 110 | 114 | 215 | 215 | 129 | 129 | 170 | 172 | 213 | 213 | 343 | 357 | 199 | 201 | 126 | 126 | 398 | 424 | 356 | 356 | 433 | 433 | 279 | 289 | 160 | 168 |
| BW_001524 | Alc | - | - | - | - | 183 | 189 | 143 | 143 | 168 | 170 | 219 | 219 | 345 | 345 | 209 | 213 | 110 | 128 | 410 | 410 | 340 | 348 | 411 | 435 | 301 | 303 | - | - |
| BW_001525 | Alc | 144 | 148 | - | - | 205 | 217 | 139 | 139 | 170 | 170 | 221 | 225 | 345 | 345 | 209 | 209 | 112 | 128 | 410 | 410 | 332 | 338 | 431 | 443 | 297 | 299 | 152 | 164 |
| BW_001526 | Alc | 146 | 148 | - | - | 207 | 213 | - | - | 170 | 176 | 219 | 227 | 343 | 345 | 211 | 211 | 126 | 126 | 408 | 412 | - | - | 433 | 433 | 291 | 297 | 158 | 166 |
| BW_F01441 | Alc | 138 | 138 | 104 | 104 | - | - | 135 | 135 | 166 | 170 | 219 | 229 | 343 | 345 | 203 | 213 | 126 | 128 | - | - | 314 | 350 | 439 | 439 | 297 | 297 | 164 | 166 |
| BW_F04444 | Alc | 152 | 154 | 102 | 102 | 203 | 207 | 131 | 143 | 176 | 176 | 249 | 249 | 359 | 367 | 209 | 209 | 118 | 118 | 412 | 420 | 322 | 348 | 429 | 433 | 289 | 301 | 162 | 168 |
| BW_FA4445 | Alc | 122 | 146 | - | - | 213 | 221 | 135 | 137 | 176 | 192 | 249 | 249 | 359 | 365 | 199 | 199 | 116 | 118 | 410 | 416 | 322 | 322 | 439 | 447 | 293 | 295 | 164 | 166 |
| BW_M01512 | Alc | 142 | 142 | - | - | 213 | 213 | 139 | 143 | 188 | 196 | 249 | 249 | 361 | 375 | 199 | 209 | 116 | 118 | 410 | 410 | 314 | 322 | 439 | 439 | 297 | 301 | 164 | 168 |
| DS_FA4469 | Alc | 152 | 154 | 92 | 92 | 213 | 213 | 135 | 143 | 194 | 196 | 249 | 249 | 361 | 361 | 201 | 215 | 118 | 120 | 410 | 410 | 314 | 338 | 439 | 439 | 293 | 301 | 164 | 166 |
| DS_M04474 | Alc | 122 | 138 | 92 | 92 | 215 | 223 | 137 | 139 | 166 | 168 | 249 | 249 | 359 | 359 | 199 | 211 | 118 | 118 | 404 | 410 | 322 | 340 | 441 | 449 | 295 | 297 | 164 | 168 |
| DS_MA4468 | Alc | 148 | 148 | 102 | 102 | 209 | 213 | 131 | 131 | 176 | 176 | 249 | 249 | 359 | 359 | 199 | 209 | 116 | 124 | 410 | 410 | 322 | 348 | 435 | 439 | 289 | 289 | 164 | 170 |
| DS_MA4470 | Alc | 124 | 142 | 92 | 92 | 207 | 217 | 133 | 139 | 176 | 176 | 225 | 227 | 361 | 381 | 199 | 213 | 118 | 118 | 408 | 408 | 324 | 340 | 433 | 443 | 295 | 301 | 166 | 168 |
| DS_MA4930 | Alc | 148 | 152 | 102 | 102 | 213 | 213 | 131 | 131 | 176 | 176 | 249 | 249 | 363 | 363 | 201 | 211 | 116 | 126 | 410 | 410 | 322 | 348 | 435 | 439 | 289 | 289 | 164 | 170 |
| DS_MJ4931 | Alc | 134 | 154 | - | - | 195 | 207 | 137 | 139 | 176 | 190 | 249 | 249 | 363 | 377 | 211 | 215 | 126 | 126 | 408 | 412 | 322 | 322 | 421 | 433 | 295 | 297 | 168 | 170 |
| PC_M04398 | Alc | 146 | 152 | 92 | 92 | 207 | 207 | 139 | 143 | 184 | 196 | 249 | 249 | 359 | 361 | 213 | 219 | 118 | 118 | 410 | 412 | 322 | 338 | 433 | 433 | 297 | 301 | 164 | 166 |
| PC_F05005 | Alc | 140 | 150 | 0 | 0 | 209 | 213 | 131 | 147 | 176 | 192 | 249 | 249 | 367 | 373 | 209 | 209 | 118 | 124 | 410 | 410 | 314 | 314 | 435 | 439 | 289 | 305 | 166 | 166 |
| PC_M04397 | Alc | - | - | 76 | 92 | 213 | 213 | 141 | 141 | 170 | 176 | 249 | 249 | 363 | 363 | 209 | 209 | 110 | 130 | - | - | - | - | 439 | 439 | 299 | 299 | 164 | 164 |
| PC_M04399 | Alc | - | - | 78 | 84 | - | - | 143 | 147 | 192 | 192 | 221 | 221 | 345 | 345 | 203 | 211 | 110 | 110 | - | - | 332 | 332 | 431 | 435 | 303 | 305 | 154 | 154 |
| PC_M05006 | Alc | 138 | 146 | 86 | 86 | 205 | 207 | 133 | 135 | 186 | 188 | 249 | 249 | 361 | 365 | 201 | 209 | 118 | 118 | 410 | 410 | 314 | 334 | 431 | 431 | 293 | 293 | 164 | 168 |
| WK_F04454 | Alc | 140 | 142 | 70 | 92 | 195 | 203 | 129 | 137 | 176 | 188 | 249 | 249 | 361 | 361 | 201 | 209 | 116 | 116 | 406 | 414 | 314 | 322 | 421 | 429 | 287 | 295 | 152 | 168 |
| WK_FA4453 | Alc | 144 | 162 | 70 | 70 | 209 | 213 | 131 | 139 | 192 | 196 | 249 | 249 | 359 | 359 | 215 | 217 | 118 | 118 | 410 | 410 | 314 | 314 | 435 | 439 | 289 | 297 | 152 | 162 |
| WK_FA5223 | Alc | 140 | 148 | 92 | 92 | 207 | 213 | 135 | 135 | 188 | 196 | 227 | 249 | 361 | 361 | 203 | 211 | 116 | 116 | 408 | 410 | 338 | 338 | 433 | 439 | 293 | 293 | 162 | 168 |
| WK_FJ5220 | Alc | 138 | 142 | 92 | 92 | 213 | 213 | 139 | 143 | 190 | 192 | 209 | 249 | 365 | 371 | 209 | 209 | 116 | 118 | 414 | 420 | 322 | 338 | 439 | 439 | 297 | 301 | 164 | 168 |
| WK_M04456 | Alc | 146 | 146 | - | - | 205 | 209 | 127 | 143 | 192 | 216 | 249 | 253 | 361 | 373 | 199 | 215 | 116 | 120 | 378 | 396 | 356 | 366 | 431 | 441 | 291 | 293 | 156 | 160 |
| WK_MA4452 | Alc | 142 | 146 | - | - | 205 | 215 | 141 | 145 | 176 | 188 | 249 | 249 | 359 | 383 | 201 | 201 | 118 | 122 | 410 | 410 | 322 | 322 | 431 | 441 | 299 | 303 | 168 | 170 |
| PS_F02873 | Alc | 146 | 152 | - | - | 213 | 213 | 117 | 141 | 186 | 190 | 217 | 225 | 345 | 345 | 201 | 215 | 130 | 138 | 410 | 420 | 314 | 322 | 439 | 439 | 275 | 299 | 166 | 168 |
| PS_M02875 | Alc | 142 | 142 | 70 | 70 | 213 | 213 | 133 | 147 | 194 | 208 | 215 | 223 | 345 | 345 | 201 | 209 | 126 | 140 | 410 | 410 | 322 | 322 | 439 | 439 | 291 | 305 | 166 | 168 |
| PS_M03375 | Alc | 144 | 144 | 92 | 92 | 205 | 213 | 115 | 145 | 186 | 186 | 249 | 249 | 361 | 373 | 201 | 209 | 116 | 118 | 404 | 412 | 322 | 340 | 433 | 439 | 275 | 303 | 164 | 166 |
| PS_M03379 | Alc | 144 | 148 | 94 | 94 | 207 | 207 | 139 | 153 | 180 | 198 | 249 | 249 | 359 | 373 | 199 | 201 | 116 | 118 | 410 | 424 | 314 | 340 | 433 | 433 | 297 | 313 | 166 | 168 |
| BS_M01530 | Alc | 146 | 148 | 86 | 92 | 213 | 219 | 139 | 139 | 170 | 174 | 225 | 229 | 343 | 345 | 211 | 213 | 128 | 138 | 410 | 410 | 314 | 338 | 439 | 445 | 297 | 297 | 152 | 162 |
| BS_F01531 | Alc | 128 | 128 | - | - | - | - | 139 | 139 | 170 | 170 | 219 | 221 | 343 | 347 | 213 | 215 | 126 | 128 | - | - | - | - | 433 | 433 | - | - | - | - |
| TR_F01442 | Alc | 150 | 150 | - | - | - | - | - | - | 170 | 192 | 217 | 227 | 345 | 345 | 209 | 209 | 110 | 140 | 410 | 410 | 348 | 348 | 439 | 439 | 283 | 283 | 154 | 154 |
| TR_M04481 | Alc | 134 | 140 | 88 | 88 | 207 | 213 | 143 | 145 | 186 | 192 | 249 | 249 | 361 | 361 | 199 | 213 | 116 | 134 | 410 | 424 | 330 | 334 | 433 | 439 | 303 | 303 | 166 | 170 |
